# EODE-PFA: A Multi-Strategy Enhanced Pathfinder Algorithm for Engineering Optimization and Feature Selection

**DOI:** 10.3390/biomimetics11010057

**Published:** 2026-01-08

**Authors:** Meiyan Li, Chuxin Cao, Mingyang Du

**Affiliations:** 1 School of Science, Hainan University, Haikou 570100, China; 2 School of Information and Communication Engineering, Hainan University, Haikou 570100, China; 3 School of Automobile and Traffic Engineering, Nanjing Forestry University, Nanjing 210037, China

**Keywords:** pathfinder algorithm, multi-strategy enhanced pathfinder algorithm (EODE-PFA), elite opposition-based learning, differential evolution algorithm, engineering optimization, feature selection, swarm intelligence optimization algorithm

## Abstract

The Pathfinder Algorithm (PFA) is a bionic swarm intelligence optimization algorithm inspired by simulating the cooperative movement of animal groups in nature to search for prey. Based on fitness, the algorithm classifies search individuals into leaders and followers. However, PFA fails to effectively balance the optimization capabilities of leaders and followers, leading to problems such as insufficient population diversity and slow convergence speed in the original algorithm. To address these issues, this paper proposes an enhanced pathfinder algorithm based on multi-strategy (EODE-PFA). Through the synergistic effects of multiple improved strategies, it effectively solves the balance problem between global exploration and local optimization of the algorithm. To verify the performance of EODE-PFA, this paper applies it to CEC2022 benchmark functions, three types of complex engineering optimization problems, and six sets of feature selection problems, respectively, and compares it with eight mature optimization algorithms. Experimental results show that in three different scenarios, EODE-PFA has significant advantages and competitiveness in both convergence speed and solution accuracy, fully verifying its engineering practicality and scenario universality. To highlight the synergistic effects and overall gains of multiple improved strategies, ablation experiments are conducted on key strategies. To further verify the statistical significance of the experimental results, the Wilcoxon signed-rank test is performed in this study. In addition, for feature selection problems, this study selects UCI real datasets with different real-world scenarios and dimensions, and the results show that the algorithm can still effectively balance exploration and exploitation capabilities in discrete scenarios.

## 1. Introduction

Complex optimization problems have long been a common research focus in both academic and industrial circles [1]. Traditional optimization methods, such as linear programming, integer programming, and dynamic programming, exhibit stable performance in simple optimization problems with low dimensions, convexity, and linear constraints. However, real-world engineering problems are mostly characterized by nonlinear constraints, high-dimensional solution spaces, non-convexity, and high computational complexity [2]. Due to their reliance on gradient information and sensitivity to problem characteristics, traditional methods tend to fall into local optima and struggle to meet the requirements of complex engineering applications. Against this background, optimization methods have gradually shifted from traditional numerical algorithms to more advanced intelligent optimization algorithms [3]. Inspired by natural phenomena, these intelligent optimization methods possess core advantages, including no requirement for gradient information, population-based collaborative search, and flexible structures. They can effectively avoid local optima and efficiently approximate the global optimal solution, thus becoming the mainstream technology for solving complex optimization problems [4].

Against this background, various bionic intelligence optimization algorithms have been constantly emerging and have demonstrated promising application prospects in fields such as engineering optimization, parameter tuning, feature selection, and path planning. For instance, the Harris Hawk Optimization (HHO) has been applied to drone systems [5], disease diagnosis [6], and optimal scheduling of microgrids [7]; the Dung Beetle Optimizer (DBO) has been used in numerical and circuit problem optimization [8], drone path planning [9], and feature selection [10]; the Snow Geese Algorithm (SGA) has found applications in biogas production prediction [11], numerical optimization problems [12], software defect prediction [13], and resource scheduling [14]; the Artificial Lemming Algorithm (ALA) has been employed in robot path planning [15], cloud task scheduling [16], and drone path planning [17]; furthermore, the Grey Wolf Optimizer (GWO) has been applied to feature selection, disease prediction [18], and student academic performance prediction [19]; the improved Sparrow Search Algorithm has been used for vehicle path planning [20] and machine learning hyperparameter optimization [21]; a novel meta-heuristic algorithm, the Advanced Social Memory Optimization (ASMO), has been utilized for numerical and engineering optimization problems [22]; and the improved Seagull Optimization Algorithm (SOA) has been applied to disease detection [23] and feature selection [24].

However, the “No Free Lunch” theorem [25] states that no algorithm can achieve optimal performance across all problems. That is to say, every meta-heuristic algorithm has certain inherent drawbacks. Therefore, researchers have proposed hybrid strategies that integrate the advantages of multiple optimization algorithms to solve complex optimization problems more efficiently. Challenged by real-world complex problems, various new hybrid algorithms have been continuously emerging [26,27]. Hybrid algorithms proposed in recent years include the hybrid genetic algorithm (GA) and simulated annealing (SA) for nurse scheduling problems [28]; the GWO-PSO-SA algorithm for solving multi-objective complex problems of electric vehicle-mounted drone inspection [29]; the hybrid GA and local search (LS) for vehicle path planning problems [30]; the Hybrid Firefly-Coyote Optimization Algorithm (HF-COA) for directional sensor network (DSN) optimization [31]; and the Teaching-Learning-Based Pathfinder Algorithm (TLPFA) for function optimization and engineering problems [32].

PFA is a swarm intelligence optimization algorithm proposed by Yapici et al. [33], which is inspired by the behavior of cooperative movement and optimization of animal groups and has the advantages of simple structure, efficient search, and easy implementation. However, through our analysis of the original PFA, three significant limitations are found to exist in it: first, the leader is only the current optimal individual, and the scarcity of the number of leaders easily causes the algorithm to fall into the local optimum and slow convergence; then the follower’s update mechanism is single and over-dependent on the leader’s position, which affects the local optimization ability in the later stage; next, the algorithm parameters mostly use fixed interval random numbers, which makes it difficult to balance the convergence speed and solution accuracy requirements.

To address the aforementioned issues, this paper proposes an enhanced Pathfinder Algorithm based on multi-strategy collaborative improvement (EODE-PFA). The core innovation lies in the design of synergistic effects among the improved strategies: first, simulate the natural law of dominant groups leading the entire population to construct an elite center, which provides a stable and efficient guiding direction for the population to resolve the one-sidedness of guidance from a single leader; second, draw on the survival strategy of leaders guiding and followers following within the population, and simultaneously improve the update mechanisms of both leaders and followers with the help of two types of mutation operators to expand the search boundary and increase population diversity; finally, dynamically control the change rhythm of the guidance mechanism and the two types of mutation mechanisms through a multi-adaptive parameter regulation mechanism. Through the collaborative cooperation among the improved strategies, the algorithm effectively balances the requirements of global exploration in the early stage and local exploitation in the later stage, avoiding the insufficiency of exploration or exploitation capabilities caused by introducing only a single strategy.

To verify the comprehensive performance of EODE-PFA, this paper applies it to the CEC2022 benchmark functions, three types of complex engineering design problems, and six sets of feature selection problems with different dimensions for testing, and compares it with eight mature intelligent optimization algorithms (including four new algorithms proposed in the past two years and four classic algorithms). The results show that EODE-PFA has significant advantages and certain competitiveness in performance indicators such as convergence accuracy and convergence speed, which fully verify the effectiveness and universality of EODE-PFA.

This paper’s core contribution is as follows:

1. Aiming at the limitations of the original PFA, an enhanced Pathfinder Algorithm with multi-strategy collaborative improvement (EODE-PFA) is proposed. Its uniqueness and advantages lie in the in-depth integration and synergistic enhancement among strategies: first, an elite group guidance mechanism is constructed, and the guidance direction is generated through the collaborative decision-making of the dominant group to solve the search yaw problem caused by the original PFA’s reliance on a single elite; then, two types of mutation operators—elite opposition-based mutation operator based on the elite center and differential mutation operator—are used to simultaneously enhance the diversity of leaders and followers; next, an adaptive parameter regulation mechanism based on iterative changes is designed, which dynamically changes according to the requirements of the early and late stages of iteration. The synergistic effect generated by the in-depth integration of the above improved strategies achieves the balance between the global exploration capability and local exploitation capability of the algorithm.
2. Construct a multi-scenario experimental system that integrates the CEC2022 benchmark function, optimization of three complex engineering design problems, and application of six feature selection tasks with different dimensions to verify the practicality, effectiveness, and universality of EODE-PFA in three scenarios: numerical optimization, engineering practice, and feature selection.
3. Through comparative experiments with eight mature intelligent optimization algorithms, the advantages and competitiveness of EODE-PFA are verified.
4. The multi-strategy collaborative improvement mechanism proposed in this paper provides an effective and transferable strategic and technical reference for intelligent optimization algorithms, enriching the improvement methods for such algorithms.

The rest of this paper is structured as follows: Section 2 presents the related work, including the basic theory of the original PFA and a relevant literature review; Section 3 elaborates on each improved strategy of EODE-PFA and analyzes its time complexity; Section 4 conducts simulation experiments on CEC2022 benchmark functions and engineering problems and carries out ablation experiments on key strategies in the benchmark function experimental scenario; Section 5 focuses on the application of EODE-PFA in feature selection problems. Finally, Section 6 summarizes the key conclusions of this paper and outlines future research directions.

## 2. Related Work

PFA is a bionic optimization algorithm that mimics the cooperative foraging behavior of animal groups. In PFA, the individual with the optimal fitness is designated as the leader, and the rest are defined as followers. Notably, the roles of leader and follower are not fixed—they can shift based on objective achievement capabilities: a leader may transition into a follower and vice versa [34,35]. In the pathfinding process, the leader’s position is regarded as the current global optimal position, toward which other individuals in the population move.

In recent years, PFA has been widely applied to solving optimization problems across diverse fields. Regarding algorithmic improvements, many researchers have developed enhancement strategies to mitigate PFA’s inherent limitations—including its proneness to local optima and inadequate population diversity.

To avoid the algorithm falling into local optima, Tang et al. [36] introduced an acceptance operator, an exchange operator, and a mutation mechanism in the follower phase of PFA. Meanwhile, a guidance mechanism was incorporated in the path exploration phase to balance exploration and exploitation capabilities. Verification on nine practical optimization problems demonstrated that its performance is superior to other algorithms; Tang et al. [32] proposed the Teaching-Learning Pathfinder Algorithm (TLPFA), which integrates the “teaching-learning” two-phase mechanism of Teaching-Learning-Based Optimization (TLBO) with the “leader-follower” mechanism of PFA. Teaching logic was introduced in the leader phase to generate a global guidance direction, and an exponential step size was designed in the follower phase combined with a learning mechanism. In mechanical engineering optimization problems, its convergence speed was 40% faster than that of the original PFA; Adegboye et al. [37] proposed the Quadratic Interpolation Hybrid Improved Pathfinder Algorithm (QHIPFA), which integrates Quadratic Interpolation (QI) technology and the Salp Swarm Algorithm (SSA) into PFA. QI was used to enhance local search accuracy, and SSA to expand population diversity. In benchmark function tests, its local optimization capability was improved by approximately 12% compared with the original PFA, making it particularly suitable for high-dimensional complex optimization scenarios; Wang et al. [38] proposed an Improved Pathfinder Algorithm (APFA) by constructing a triple improvement framework of “multi-elite guidance + Logistic chaotic mapping + comprehensive following”. Multiple elite individuals were used to replace a single leader to avoid local optima, chaotic mapping was applied to initialize the population to improve diversity, and a comprehensive following strategy was adopted to balance the intensity of individual interaction. The Improved Pathfinder Algorithm (IPFA) proposed by Mao et al. [39] integrates elite opposition-based learning and Grey Wolf Optimization (GWO), achieving significantly improved solution accuracy in complex engineering optimization problems such as pressure vessel optimization and extension spring optimization. In terms of parameter and encoding optimization, Mahapatra et al. [40] proposed ASDR-PFA based on Adaptive Search Dimension Ratio (ASDR), which improves convergence speed by dynamically updating the SDR parameter. Ademola et al. [41] introduced inertia weight into PFA (IPFA), realizing the reduction of active power loss and the improvement of voltage profile; Li et al. [42] adopted Discrete Complex-Valued Encoding (DCPFA) for PFA, which was applied to wind farm layout optimization and achieved the optimal unit power cost under two complex scenarios; Li et al. [43] incorporated quantum computing into PFA (QPFA), improving the parameter extraction accuracy of Proton Exchange Membrane Fuel Cells (PEMFCs); in the direction of multi-objective optimization, Li et al. [44] proposed MOPFA based on elite dominance and crowding distance, which was used for multi-objective optimal power flow problems containing renewable energy, generating a uniformly distributed Pareto front; Jiang et al. [45] improved the multi-objective PFA (IMOPATH), combining the Elman neural network to realize power load interval prediction with a higher prediction interval coverage probability and a narrower width.

In terms of application scope, PFA and its modified versions have found extensive utility in production scheduling, energy systems, data processing, and other domains. Within production scheduling, Dong et al. [46] put forward the Improved Discrete Prairie Dog Optimization Algorithm (IDPFA) to address the green flow shop scheduling problem constrained by limited buffers and Automated Guided Vehicles (AGVs). To this end, they designed three crossover operations to replace the conventional exploration phase and introduced a multi-neighborhood local search strategy, thereby realizing dual-objective optimization of minimizing the maximum makespan and total energy consumption. Wang Qingzheng et al. [38] focused on the flexible job shop scheduling problem with restricted transportation resources, designing an initialization method that accounts for continuous transportation and a local search strategy with multiple neighborhood structures. This work provides a valuable reference for the scenario adaptation of PFA in subsequent applications related to the collaborative optimization of transportation resources in flexible job shop scheduling. In the field of energy systems, Pan et al. [47] proposed a multi-objective discrete allocation PFA to construct a renewable energy consumption optimization model, and the proposed algorithm outperformed the multi-objective multiverse optimizer and the Sparrow Search Algorithm by 9.54% and 14.67% in fitness values, respectively, with an average power grid simulation efficiency of 96.16%; Varaprasad [48] applied PFA to achieve the optimal configuration of photovoltaic systems in multi-side distribution networks, significantly reducing power loss; Gouda et al. [49] utilized PFA to study the dynamic performance of fuel cells, and the constructed Simulink dynamic model could accurately analyze the response to load changes. In the field of data processing and fault diagnosis, Huang et al. [50] combined PFA with the Gaussian Mixture Model (PFA-GMM) to automatically determine the number of clusters, achieving better clustering performance than comparative algorithms on both synthetic and real datasets; Halder et al. [51] proposed an Enhanced PFA (EPFA) combined with Motor Current Signal Analysis (MCSA) to detect rotor bar breakage faults in induction motors, overcoming the limitations of traditional frequency domain analysis; Zhou et al. applied PFA to detect fault pulses in original signals, verifying its effectiveness in signal processing. In other fields, Adnane et al. [52] combined opposition-based learning with PFA (OBLPFA) to optimize the execution time, cost, and resource utilization of workflow scheduling in cloud environments; Dogan et al. [53] proposed a Binary PFA (BPFA) for the optimization of bus route segmentation problems; Xiangbo et al. [54] incorporated the differential evolution mutation operator into PFA (HPFA), which exhibited superior performance over comparative algorithms in data clustering and engineering design problems; Yapici [55] improved PFA (mPFA) for optimal reactive power dispatch problems.

Among the aforementioned PFA variants, many are based on a single improved strategy. For example, ASDR-PFA [40] only introduces the parameter optimization strategy of “Adaptive Search Dimension Ratio (ASDR)”; DCPFA [42] merely modifies PFA into a discrete complex-valued form without additional improved strategies; the binary variant BPFA [53] only adapts to binary encoding without other strategy enhancements; HPFA [54] only integrates the differential evolution (DE) mutation operator and does not combine other improvement mechanisms. A single improved strategy cannot simultaneously meet the requirements of comprehensive exploration and local optimization. Compared with these PFA variants, the core advantage of the proposed EODE-PFA lies in the synergy of multiple strategy fusion: for instance, although IPFA [39] introduces EOBL, it lacks the group collaborative guidance of an elite center, leading to mutations that tend to fall into local regions; HPFA [54] only integrates DE mutation without optimizing the leader guidance mechanism and dynamic parameter regulation, resulting in insufficient global exploration; OBLPFA [52] only introduces the opposition-based learning strategy alone, without the coordination of a guidance mechanism and dynamic parameter regulation, leading to easy yaw of the search direction and limited convergence efficiency. In contrast, EODE-PFA first provides a precise direction for subsequent mutations through the guidance mechanism, then expands the search range via dual mutation operators, and finally dynamically adapts to the requirements of different iterative stages through the parameter mechanism. These improved strategies cooperate with each other and exert synergistic effects, which is also the key reason for their excellent performance in multi-scenario tests.

Next, we will elaborate on the basic theory of the original PFA.

In the Pathfinder Algorithm, the individual with the optimal fitness is designated as the leader to perform a global search for exploring new regions, while the remaining individuals act as followers to complete local optimization by following the leader’s position with reference to their own awareness. The algorithm is thus divided into two pathfinding phases:

The first phase is the leader exploration phase. For the leader (denoted as $x_{p}$), the update formula for its position is as follows:

(1) $$x_{p}^{K+1}=x_{p}^{K}+2r_{3}\cdot (x_{p}^{K}-x_{p}^{K-1})+A$$

where $x_{p}^{k+1}$ denotes the updated position vector of the leader, $x_{p}^{k}$ represents the current position vector of the leader, and $x_{p}^{k-1}$ stands for the position vector of the leader at the previous moment. *K* is the current iteration number, and $r_{3}$ is a randomly generated vector within the interval [0,1]. *A* is the random step size of the path explorer, whose magnitude is determined by the multi-directionality parameter of $u_{1}$. The value of *A* is calculated via Equation (4) in each iteration.

The second phase is the follower exploitation phase. All followers adopt the update method described in Equation (2) to move toward the leader, as detailed below:

(2) $$x_{i}^{K+1}=x_{i}^{K}+R_{1}\cdot (x_{j}^{K}-x_{i}^{K})+R_{2}\cdot (x_{p}^{K}-x_{i}^{K})+\varepsilon ,i\geq 2$$

where *K* is the current iteration number; $x_{i}$ and $x_{j}$ respectively represent the position vectors of the *i*-th follower and the *j*-th follower; $x_{i}^{k+1}$ denotes the updated position vector of the *i*-th follower; and $x_{p}^{k}$ is the position vector of the leader. Specifically, $R_{1}=\alpha r_{1}$ and $R_{2}=\beta r_{2}$, where $r_{1}$ and $r_{2}$ are uniformly generated random variables within the interval [0,1]—thus, $R_{1}$ and $R_{2}$ are random vectors. Here, *α* is the interaction coefficient, representing the movement range of an individual relative to its neighbors; *β* is the attraction coefficient, determining the distance between an individual and the leader. As indicated in the original literature [55], α and *β* are randomly selected from the range [1,2] during iterations. Meanwhile, *ε* is a coefficient that adds randomness to followers and is generated via Equation (3) in each iteration:

(3) $$\varepsilon \;=\left(1-\frac{K}{K_{\max}}\right)\cdot u_{1}\cdot D_{\mathrm{ij}},{\;D}_{\mathrm{ij}}\;=\;\|x_{i}-x_{j}\|$$

(4) $$A=u_{2}\cdot e^{\frac{-2K}{K_{\max}}}$$

where $u_{1}$ and $u_{2}$ are random vectors within the range [−1, 1], which are used to influence the movement step size of individuals. These vectors also enable individuals to move back to their previous positions. However, if $u_{1}$ and $u_{2}$ exceed this range, individuals will undergo significant position adjustments, thereby deviating from potential solutions. $D_{ij}$ represents the distance between two members, and *K_max_* denotes the maximum iteration number.

The pseudo-code of the Pathfinder Algorithm is presented in Algorithm 1.
**Algorithm 1**: Pseudo-code of PFA1.  Load PFA parameter 2.  Initialize the population 3.  Calculate the fitness of initial population 4.  Find the pathfinder 5.  **While**
*K* < maximum number of iterations 6.        *α* and *β* = random number in [1,2] 7.        Update the position of pathfinder using Equation (1) and check the bound 8.        **if** new pathfinder is better than old 9.               Update pathfinder 10.       **end** 11.       **for**
*i* = 2 to maximum number of populations 12.             Update the position of members using Equation (2) and check the bound 13.       **end** 14.       Calculate new fitness of members 15.       Find the best fitness 16.       **if** best fitness < fitness of pathfinder 17.             Pathfinder = best member 18.             Fitness = best fitness 19.       **end** 20.       **for**
*i* = 2 to maximum number of populations 21.             if new fitness of member(*i*) < fitness of member(*i*) 22.                 Update members 23.             **end** 24.       **end** 25.       Generate new *A* and *ε*. 26. **end**

## 3. EODE-PFA

The improved strategies of the EODE-PFA algorithm are as follows: the elite strategy and the guidance mechanism of the elite center, the elite opposition-based learning mutation operator based on the elite center (abbreviated as ECOBL), the differential mutation operator, and the iteration-based collaborative adaptive parameter regulation. These improved strategies will be elaborated on one by one in the following sections.

### 3.1. Motivation for Improvement

Despite the advantages of PFA, including simple structure, high operability, and efficient problem-solving capability, three significant limitations are identified in the algorithm: ① The leader is only the current optimal individual, and the scarcity of leaders is prone to cause the algorithm to fall into local optima and lead to slow convergence; ② The follower update mechanism is singular, with excessive reliance on the leader’s position, which impairs the local optimization capability in the later stage; ③ Most parameters adopt random numbers within fixed intervals, making it difficult to balance the requirements of convergence speed and solution accuracy. To address these issues, this paper proposes an enhanced PFA based on multi-strategy collaborative improvement, named EODE-PFA. The improved strategies can be summarized as one guidance mechanism, two types of mutation operators, and one iteration-based adaptive parameter regulation mechanism, which are specified as follows:

1. A hybrid mechanism of “guidance + mutation” is designed for the leader phase. With the guidance of the elite strategy and elite center as well as the ECOBL mutation operator, it can not only improve the diversity of the leader population but also explore high-quality potential solutions.
2. An iteration-based adaptive parameter regulation mechanism is constructed to dynamically balance the requirements of prioritizing exploration in the early stage and prioritizing local optimization in the later stage.
3. The differential mutation operator is introduced in the follower phase. Drawing on the core operations of “mutation, crossover and selection” in the differential evolution algorithm, the update mechanism is improved to enhance the diversity of the local search space.
4. The following section will sequentially introduce the improved strategies and time complexity analysis of EODE-PFA.

These strategies are not simply superimposed or function independently; rather, they implement targeted collaborative optimization aiming at the core defects of the original algorithm. To address the issue that the leader consists only of the current single optimal individual, which is prone to falling into local optima, through the organic combination of the guiding role of the elite strategy and the ECOBL mutation operator expands the diversity of the leader population is expanded to ensure the continuity of high-quality solutions. On the other hand, it explores the potential high-quality solution space. For the problem that the follower update mechanism is overly dependent on the leader, a differential mutation operator is introduced to broaden the local search paths. Meanwhile, with the help of an adaptive parameter adjustment mechanism that dynamically adjusts with iterations, such collaborative operation fundamentally balances the global exploration and local exploitation capabilities, achieving the synchronous improvement of convergence speed and optimization accuracy.

### 3.2. Elite Strategy Guidance Mechanism

The leader update mechanism shows that PFA only uses a single individual with the best fitness as a leader. This mechanism has obvious limitations: the number of leaders is scarce, and the update structure is simple, which easily leads to the insufficient exploration capability of the algorithm and then causes the convergence stagnation and local optimum problems.

To address this issue in the EODE-PFA algorithm, all individuals are first sorted according to their initial fitness values, and a subset of individuals with the optimal fitness is redefined as the leader group. Through extensive comparative experimental analysis and considering that the leaders need to be representative while their number should not be excessive, the top 10% of individuals are selected here as the leader group. Meanwhile, to ensure that a sufficient number of leaders can be obtained to construct a stable collaborative guidance system even in small-scale population scenarios, and to avoid the tendency to fall into local optima caused by a single leader or a very small number of leaders, a lower limit of 5 is set for the size of the leader group. Instead of relying on a single best individual, the arithmetic average position of these leaders (denoted as the “elite center” $X_{EC}$) is then used to guide the movement of the group together. This improvement can enhance the global search ability of the algorithm and increase the exploration speed. In this paper, the elite is the leader, and the elite center is calculated as follows.

(5) $$X_{EC}=\frac{1}{LN}\sum_{i=1}^{LN}x_{i}$$

where *LN* denotes the number of leaders (elites), $x_{i}$ represents the position vector of the i-th leader, and $X_{EC}$ is the position vector of the elite center.

### 3.3. Elite Opposition-Based Learning Mutation Operator Based on Elite Center (ECOBL)

Opposition-based learning (OBL) [56] was put forward by Tizhoosh in 2005, with the aim of improving the solution quality and convergence rate of intelligent optimization algorithms. This seamless integration of forward and reverse search overcomes the constraints of a single search direction, allowing the algorithm to expand its search range and thus converge rapidly toward the global optimal solution. In this context, the definition of the opposition point is given as follows:

**Definition 1.**  *Opposition point. Given a point* $x=(x_{1},x_{2},\ldots ,x_{d})$ *in a d-dimensional space, where* $x_{i}\in [lb,ub]$ *with ub an lb representing the upper and lower bounds of the search range, for* i∈[1,b] *, the opposite point* $x^{\prime }=(x_{1}^{\prime },x_{2}^{\prime },\ldots ,x_{d}^{\prime })$ *can be defined as follows:*

(6) $$x_{i}^{\prime }=lb+ub-x_{i}$$

On the basis of opposition-based learning, elite opposition-based learning (EOBL) [57] has been further proposed, which optimizes and expands the basic opposition-based learning framework by introducing the elite strategy. Compared with the solutions generated by ordinary OBL, EOBL is more likely to fall into the high-quality potential optimal solution region. Based on this, elite reverse points are defined as follows.

**Definition 2.**  *Elite opposition-based solutions. Let the elite individuals in the population be denoted as* $x_{i,j}=(x_{i,1},x_{i,2},\ldots ,x_{i,d})(i=1,2,\ldots ,N)$ *, where d represents the dimension. The elite opposition-based solution, denoted as *$x_{i,j}^{*}=(x_{i,1}^{*},x_{i,2}^{*},\ldots ,x_{i,d}^{*})$*, is defined as follows:*

(7) $$x_{i,j}^{*}=k\times \left(lb_{j}+ub_{j}\right)-x_{i,j}$$

*where* k ∈ [0,1] *of random numbers.*

Currently, elite opposition-based learning (EOBL) has been widely adopted in research focused on enhancing classical intelligence optimization algorithms, such as Particle Swarm Optimization (PSO), genetic algorithm (GA), and Grey Wolf Optimizer (GWO). As a case in point, Wang et al. [58] incorporated EOBL into the Sparrow Search Algorithm, which served to improve both the diversity and quality of the population. Lei Chen et al. [59] adopted EOBL to refine the Sine Cosine Algorithm (SCA). Additionally, EOBL was introduced into the Equilibrium Optimization Algorithm (EOA) in [60] to strengthen its global search capability. For the sake of concise expression, elite opposition-based learning solutions will be referred to as “elite opposition solutions” in the subsequent sections of this paper.

After constructing the leader group via the elite strategy, this paper combines the elite center with EOBL to derive the ECOBL mutation operator. Subsequently, this operator is employed to improve the update mechanism of the leaders, thereby enhancing the population diversity of the leader group and strengthening the algorithm’s ability to escape from local optima.

For the convenience of expression, the elite opposition-based solution based on the elite center will be referred to as the elite opposition solution in the subsequent sections of this paper.

Different from the original PFA and the original elite opposition-based learning, the elites selected in this paper are a group of a certain size rather than a single individual. Under the guidance mechanism of multiple elite individuals, the exploration speed of the population can be accelerated, and the convergence time can be shortened. Next, we present the generation formula of the elite opposition solution:

(8) $$x_{p}^{*}=X_{EC}+k\times \;\left(X_{EC}-x_{p}\right)$$

where *k* is a random number within the interval [0,1]; $x_{p}$ represents the position of the *p*-th elite individual currently to be optimized.

For each leader $x_{p}$, an elite opposition solution $x_{p}^{*}$ is generated using the update method in Equation (8) based on the elite center. For followers, instead of only following a single leader as before, they now move toward the direction of the elite center—this avoids the limitation of the group’s search range caused by the single leader’s tendency toward local optimality. The specific update formula for followers is as follows:

(9) $$x_{i}^{K+1}=x_{i}^{K}+R_{1}\cdot (x_{j}^{K}-x_{i}^{K})+R_{2}\cdot (X_{EC}^{K}-x_{i}^{K})+\varepsilon ,i\geq 2$$

where $X_{EC}$ denotes the elite center of the top 10% of individuals with the optimal fitness in the population during the current iteration.

In the next step, each elite individual $x_{p}$ generates a basic updated solution $x_{p}^{base}$ using Equation (1) from the original PFA. Subsequently, a greedy selection strategy is adopted to update the elite group, which is specified as follows:

(10) $$x_{p}^{*}=\left\{\begin{array}{ll} x_{p}^{*} & ,\;f\left(x_{p}^{*}\right)\leq f\left(x_{p}^{\mathrm{base}}\right);\\ x_{p}^{\mathrm{base}} & ,f\left(x_{p}^{*}\right)>f\left(x_{p}^{\mathrm{base}}\right);\; \end{array}\right.$$

where $f\left(x\right)$ is the fitness function for the minimization problem; $x_{p}^{*}$ denotes the elite opposition solution of the elite individual $x_{p}$; $x_{p}^{\mathrm{base}}$ represents the basic updated solution of the elite individual $x_{p}$; and $x_{p}^{*}$ is the individual selected and retained finally.

### 3.4. Differential Mutation Operator

Differential evolution (DE) is a classic intelligent optimization algorithm proposed by R. Storn and K. Price in 1995. It is developed on the basis of genetic algorithms and other evolutionary algorithms and has the characteristics of simplicity and efficiency [61]. The algorithm has been widely used to solve complex real-world optimization problems due to its advantages of fast convergence, few adjustable parameters, and a wide application range. Dou et al. [62] introduced the differential evolution algorithm to optimize the Coyote optimization algorithm (COA), forming the hybrid algorithm DECOA, which improved the parameter estimation accuracy of photovoltaic cells and the operational efficiency of the algorithm. Literature [63] integrates differential evolution algorithm optimization into the crayfish algorithm to improve the quality of color multi-threshold image segmentation.

**Figure 1:**
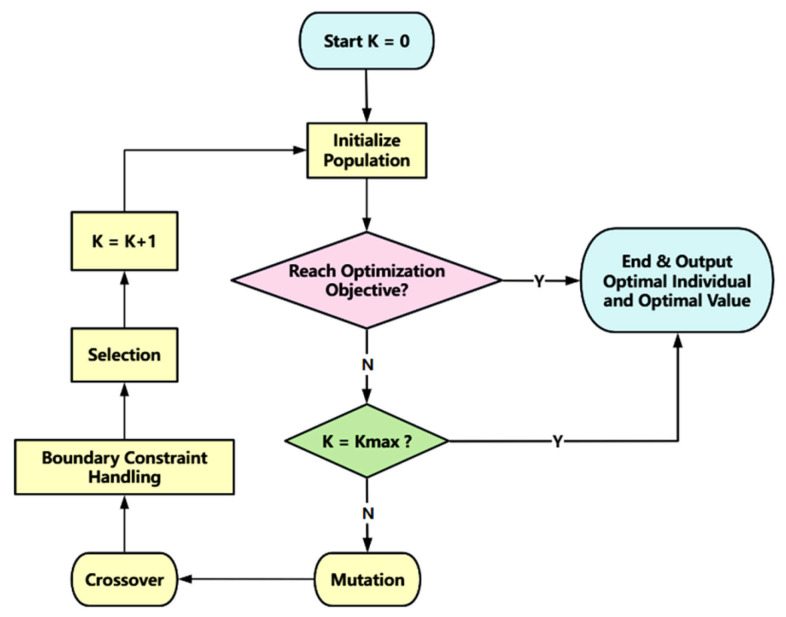
Flowchart of differential evolution algorithm.

“mutation+cross+selection” is the core operation of the algorithm, and the basic process is shown in Figure 1.

In PFA, the follower’s forward direction mainly depends on the leader and lacks the ability to explore autonomously. This easily leads to the follower blindly following the leader, which affects the local development ability of the algorithm in the later stage. In view of the fact that the differential evolution algorithm has the characteristics of fast convergence speed and strong development ability, we introduce the core operation in the follower stage to generate new candidate solutions. In the mutation phase, there are two key parameters: the mutation factor F and the crossover probability factor $cr_{i}$. R. Storn and K. Price pointed out in [61] that the recommended initial value of F is 0.5, with its effective value range generally falling between 0.4 and 1.0; the initial value of $cr_{i}$ can be set to 0.1, and they also indicated that a relatively large $cr_{i}$ value usually accelerates convergence. Through extensive comparative experimental analysis, and to balance the stability and diversity of the algorithm, both parameters are set to 0.5 in this paper. The mutation formula adopted is as follows [61]:

(11) $$V_{i}=X_{r_{1}}+F\cdot (X_{r_{2}}-X_{r_{3}})$$

where $X_{r1}$, $X_{r2}$, and $X_{r3}$ are individuals randomly selected from the entire population, with $r_{1}\neq r_{2}\neq r_{3}$. The mutation factor *F* = 0.5, and $V_{i}$ denotes the mutation vector of the *i*-th follower.

Next, the mutation vector and the current follower position undergo a crossover operation to generate the first candidate solution, i.e., the trial vector $U_{i,j}$. The binomial crossover method is adopted in this study, with the specific formula given as follows:

(12) $$U_{i,j}=\left\{\begin{array}{ll} V_{i,j}, & if\;rand<cr_{i}\;or\;j=j_{rand}\\ X_{i,j}, & else \end{array}\right.$$

Here, $U_{i,j}$ denotes the *j*-th component of the trial vector for the *i*-th particle, $V_{i,j}$ is the *j*-th component of the mutation vector for the *i*-th particle, and $X_{i,j}$ represents the *j*-th component of the current position of the *i*-th particle. ${cr}_{i}$ is the crossover probability, which is fixed at 0.5. $j_{rand}$ is a randomly selected index to ensure that at least one dimension of the mutation vector $V_{i,j}$ is utilized.

Subsequently, the algorithm generates the second candidate solution, i.e., the base update vector $x_{i}^{\mathrm{base}}$, using the update Equation (2) for followers in PFA. A greedy selection strategy is adopted to update the followers by comparing the fitness values of the two candidate solutions, with the update formula given as follows:

(13) $$x_{i}^{*}=\left\{\begin{array}{ll} U_{i} & ,\;f\left(U_{i}\right)\leq f\left(x_{i}^{\mathrm{base}}\right);\\ x_{i}^{\mathrm{base}} & ,f\left(U_{i}\right)>f\left(x_{i}^{\mathrm{base}}\right);\; \end{array}\right.$$

where $x_{i}^{*}$ denotes the individual finally selected and retained.

### 3.5. Adaptive Parameter Regulation Mechanism

Most parameters of the original PFA are randomly assigned within fixed intervals, failing to balance the global exploration in the early stage and local exploitation in the later stage. In contrast, the key parameters of EODE-PFA are dynamically adjusted with the number of iterations to meet the requirements of balancing global exploration and local exploitation. Specifically:

In the leader phase, an iterative positive increment strategy is adopted to dynamically modify the leader inertia coefficient $r_{3,}$ aiming to reduce its dependence on historical positions in the early iterations for enhanced capability of exploring new regions and to strengthen convergence toward optimal regions in the later iterations for improved local exploitation performance. To enable followers to balance the guidance from leaders and independent local exploration, an iterative positive increment strategy is applied to the interaction coefficient $r_{1}$ and an iterative reverse decrement strategy to the guidance coefficient $r_{2}$ in the follower phase, thereby balancing the impact of information interaction between leaders and other followers on the movement of current followers. The update formulas for the key parameters are given as follows:

(14) $$r_{3}=k\cdot e^{\frac{2K}{K_{\max\;}}}$$

(15) $$r_{2}=1+k\cdot e^{\frac{-2K}{K_{\max}}}$$

(16) $$r_{1}=1+k\cdot e^{\frac{2K}{K_{\max}}}$$

where *k* is a random number within the interval [0,1].

### 3.6. Time Complexity Analysis of EODE-PFA

The time complexity of EODE-PFA is mainly reflected in the processes of population initialization, leader update, and follower update. In the initialization phase, assuming the population size is N and the problem dimension is D, the time complexity of EODE-PFA is O(N × D). In the iterative process, the time complexity is O(N × *f* × T), where *f* denotes the number of independent runs and T denotes the number of iterations. In the leader update phase, the population needs to update L leaders in each iteration. Since L is a fixed constant that does not affect the order of complexity growth, the total time complexity of this phase is O(L × D × T) = O(D × T).

In the follower update phase, there are N−L followers in each iteration. According to Equation (2), the position update of followers depends on the single leader with the current optimal fitness. Since N−L is a fixed constant, the total time complexity of this phase is O((N − L) × D × T) = O(D × T).

As the overall structure of the algorithm is executed in a cascaded manner, and the total time complexity of both the leader phase and the follower phase is O(D × T), the total time complexity of EODE-PFA is derived as follows:

O(EODE-PFA) = O(N × D) + O(N × ***f*** × T) + O(D × T) = O(N × f × T) + O(N × D × T) = O(N × T × (***f*** + D)) = O(N × T × D).

This is because *f* remains a fixed constant in this study. It can be concluded that the time complexity order of EODE-PFA is consistent with that of the original Pathfinder Algorithm, and the proposed improvement methods do not alter the growth trend of the core time complexity.

The pseudo-code of EODE-PFA is presented as Algorithm 2.
**Algorithm 2**: Pseudo-code of EODE-PFA1. Load EODE-PFA parameter 2. Initialize the population 3. Calculate the fitness of initial population 4. Find the number of pathfinders(leaders): LN 5. **While** K < maximum number of iterations 6.     Calculate elite center using Equation (5) and the bound of leaders 7.     Update parameters: *u*_1_, *u*_2_, *r*_3_, *A*, *ε* 8.     **for**
*i* = 1 to LN 9.            Generate elite opposition-based position using Equation (8) 10.          Generate basic update position using Equation (1) 11.          Check the bound of elite opposition-based position 12.          **if** elite opposition-based position or basic update position is better than old 13.          Update pathfinder 14.          **end** 15.   **end** 16.   **for**
*i* = LN + 1 to maximum number of populations 17.          Generate 3 random individuals 18.          Generate mutation vector using Equation (11) 19.          Generate trial vector using Equation (12) 20.          Generate basic update position using Equation (2) 21.          **if** trial vector or basic update position is better than old 22.          Update follower 23.          **end** 24.   **end** 25.   Generate new fitness of each members **26. end**

## 4. Experimental Results and Discussion

In this section, two groups of experiments are carried out to conduct an in-depth investigation and analysis of the performance of the proposed EODE-PFA. The experimental settings encompass the CEC2022 benchmark functions and three complex engineering design optimization problems. All experiments were simulated on a personal computer equipped with an Intel(R) Core(TM) i5-10210U CPU @ 1.60 GHz and the Windows 11 operating system, and the simulation was implemented in MATLAB R2024b.

### 4.1. Tests on CEC2022 Benchmark Functions

#### 4.1.1. Exploitation and Exploration Analysis

In this section, the performance of EODE-PFA is verified using the CEC2022 benchmark functions [64]. The CEC2022 benchmark functions consist of 12 single-objective test functions, which can be categorized into four types: unimodal function (F1), basic functions (F2–F5), hybrid functions (F6–F8), and composite functions (F9–F12). All test functions are designed to solve minimization problems. Information such as the expressions, theoretical minimum values, and value ranges of the benchmark functions is presented in Table 1.

Reference [64] indicates that the CEC2022 benchmark functions support three test dimension settings: 2, 10, and 20 dimensions. Considering the complexity of the experiments, 20 dimensions are selected for the experiments in this section. The experiments are based on 30 independent runs, with 400 iterations per run. The search boundary is set to [−100, 100], and the population size *N* is uniformly set to 100 (consistent across all algorithms). Finally, the average fitness is used to plot the convergence curves.

To better highlight the advantages of the proposed algorithm, EODE-PFA is compared with four novel intelligence optimization algorithms and four mature intelligence optimization algorithms, namely, Pathfinder Algorithm (PFA, 2019) [33], Artificial Lemming Algorithm (ALA, 2025) [65], Advanced Social Memory Optimization (ASMO, 2025) [22], Sequoia Optimization Algorithm (SequoiaOA, 2025) [66], Snow Geese Algorithm (SGA, 2024) [67], Harris Hawk Optimization (HHO, 2019) [68], Grey Wolf Optimizer (GWO, 2013) [69], and differential evolution (DE, 2006) [61].

#### 4.1.2. Performance Evaluation and Convergence Curve Analysis

The statistical results of all algorithms on the CEC2022 test suite are presented in Table 2, which details the best (optimal value), worst (worst value), mean (average value), Std (standard deviation), and average running time (time/s) of each algorithm across the 12 benchmark function tests. To intuitively demonstrate the performance of EODE-PFA, the minimum value of each statistical indicator in the table is highlighted in bold.

As indicated in Table 2, EODE-PFA achieves the minimum mean value across all 12 benchmark functions, demonstrating stable and excellent overall optimization performance. Regarding the best indicator, EODE-PFA performs optimally on all functions except F5, F7, and F11: the optimal value of F5 is obtained by the SequoiaOA, that of F7 by the ALA, and that of F11 by the original PFA. In terms of the worst indicator, EODE-PFA also exhibits outstanding performance, failing to achieve the minimum value only on F3 and F12. Although EODE-PFA did not achieve the optimal results in the single indicator of best or worst for a small number of test functions, its average optimization accuracy is significantly superior to that of other comparative algorithms, which is sufficient to prove that EODE-PFA has strong comprehensive competitiveness.

In addition, although the running time of EODE-PFA is slightly longer than that of some comparative algorithms, the time gap with all algorithms is not significant, and there is no order-of-magnitude increase. Meanwhile, this is traded off for the optimal performance in the solution accuracy of the objective function: the minimum values are achieved in all cases for the mean indicator, which indicates that the overall optimization performance is superior to that of other algorithms.

**Figure 2:**
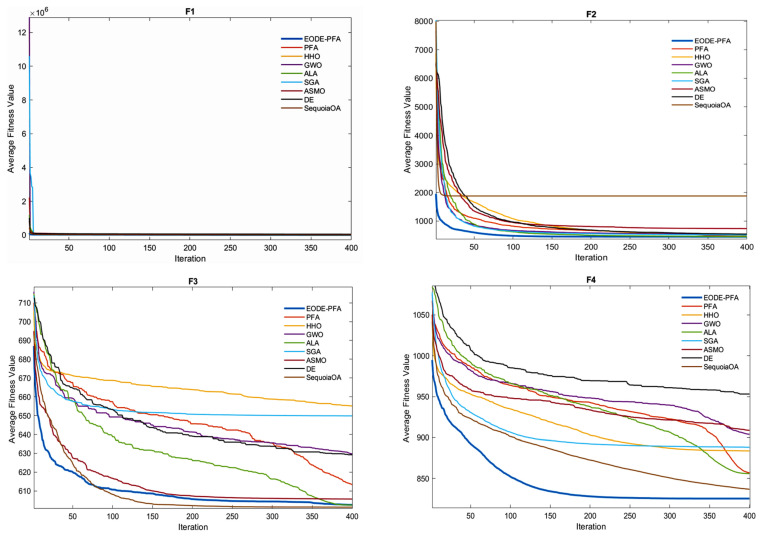

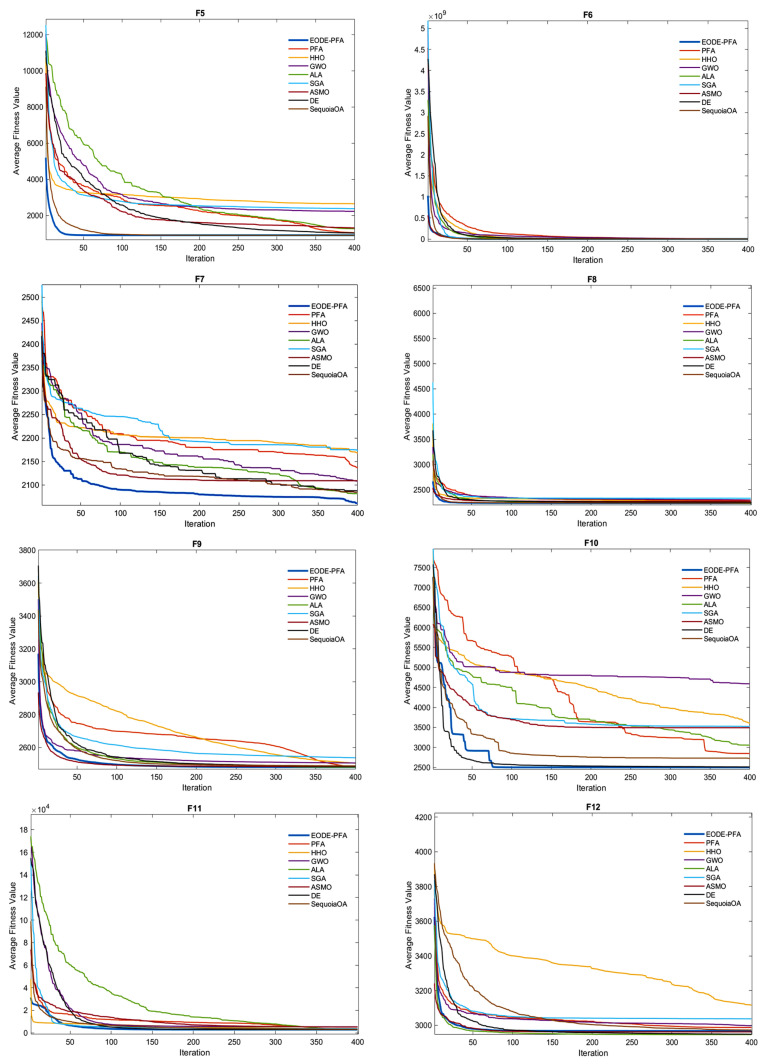
Convergence curves for CEC2022 functions optimized.

The aforementioned results demonstrate that the EODE-PFA, improved through the collaboration of multiple strategies, exhibits significantly superior optimization performance compared to the original PFA and DE, which further verifies the effectiveness and rationality of the proposed improvement strategies. Figure 2 presents the convergence curves of the test functions. It can be concluded from Figure 2 that under the test condition of 400 iterations, EODE-PFA outperforms over 90% of the comparative algorithms in both convergence speed and solution accuracy.

#### 4.1.3. Wilcoxon Signed-Rank Test of EODE-PFA

The Wilcoxon *p*-value test [70] serves as a crucial approach for verifying the presence of a statistically significant discrepancy between two data sets, with the *p*-value acting as its core metric. The *p*-value exhibits a negative correlation with the reliability of outcomes: when *p* < 0.05, it can be concluded that a notable difference exists between the data of the two algorithms, which signifies that the result holds statistical significance.

With EODE-PFA as the benchmark, the Wilcoxon signed-rank test was further employed in the experiments for evaluation, with the statistical results detailed in Table 3.

The results in Table 3 indicate that the *p*-values are less than 0.05 for most test functions. For a small number of functions, the *p*-values between EODE-PFA and individual algorithms are greater than 0.05; for instance, in F7, the *p*-value between EODE-PFA and ALA is 0.5503, and that between EODE-PFA and ASMO is 0.7369. This demonstrates that the differences between EODE-PFA and these algorithms on the corresponding functions are not significant, meaning that the performance of individual algorithms is comparable to that of EODE-PFA on certain functions. In addition, the R+ values of EODE-PFA are significantly larger than the R− values when compared with most algorithms; cases where R+ is close to or smaller than R− only occur in the comparison with individual algorithms on a few functions. This shows that EODE-PFA can stably obtain a larger advantageous rank sum on most test functions, and only in a few scenarios does its rank sum distribution approach or become inferior to that of individual algorithms, which further verifies the performance competitiveness of EODE-PFA. The results of the sign indicator show that the number of scenarios where EODE-PFA demonstrates advantages is far greater than that of non-advantageous scenarios. Its overall performance competitiveness is not affected by a small number of local non-advantageous cases, which reflects its core advantage position.

Overall, EODE-PFA exhibits significantly superior performance to the comparative algorithms on most CEC2022 test functions. This conclusion fully validates the competitiveness of EODE-PFA in complex numerical optimization tasks.

#### 4.1.4. Ablation Experiments

To verify the independent contributions and synergistic effects of the key improved strategies in EODE-PFA on the algorithm’s optimization performance, ablation experiments are conducted in this section. By comparing the performance differences between the complete algorithm and variants with a single improved strategy removed, the necessity and rationality of each improved strategy are validated.

Three ablation objects are constructed by removing three types of key strategies, namely the elite strategy, ECOBL mutation operator, and differential mutation operator:

(1) PFA1: The elite strategy in EODE-PFA is removed, while the rest of the structure and parameters remain consistent.
(2) PFA2: The ECOBL mutation operator in EODE-PFA is removed, while the rest of the structure and parameters remain unchanged.
(3) PFA3: The differential mutation operator in EODE-PFA is removed, while the rest of the structure and parameters remain unchanged.

The ablation experiments share the same experimental environment as the CEC2022 benchmark function tests, and all key parameters are consistent with those in the previous sections to ensure the consistency of the test scenario. Performance differences only result from the lack of strategies rather than changes in test conditions. The experimental evaluation metrics are also consistent with the previous sections, including mean fitness, best fitness, worst fitness, and convergence speed, while the statistical results of 30 independent runs are recorded.

**Figure 3:**
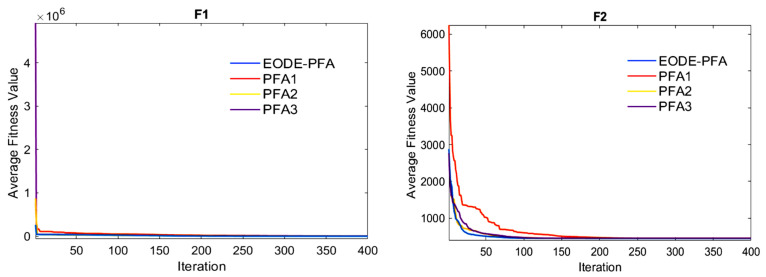

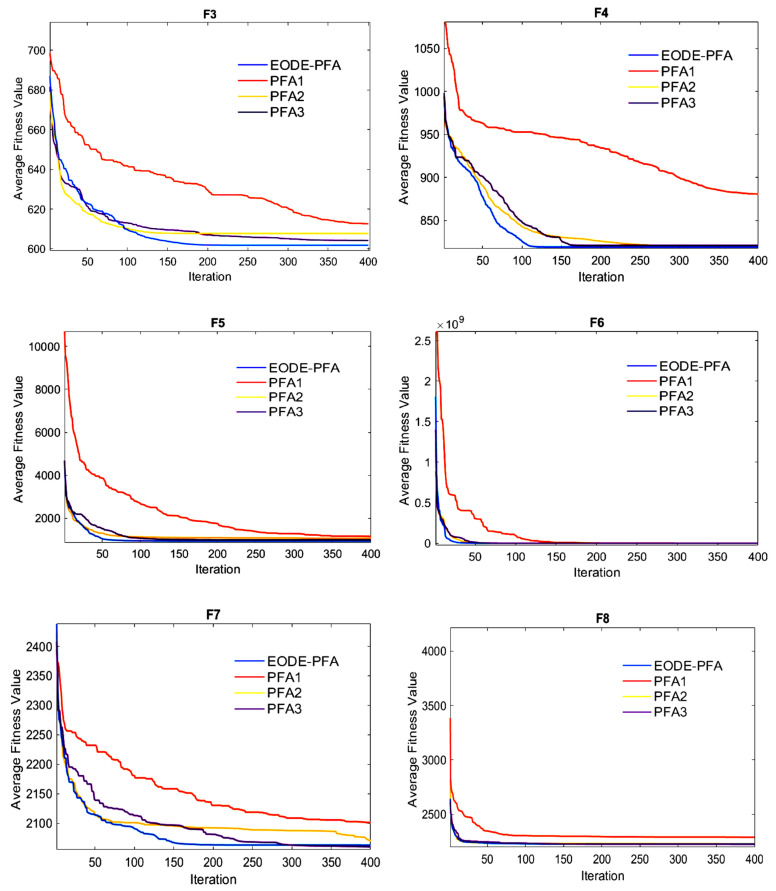

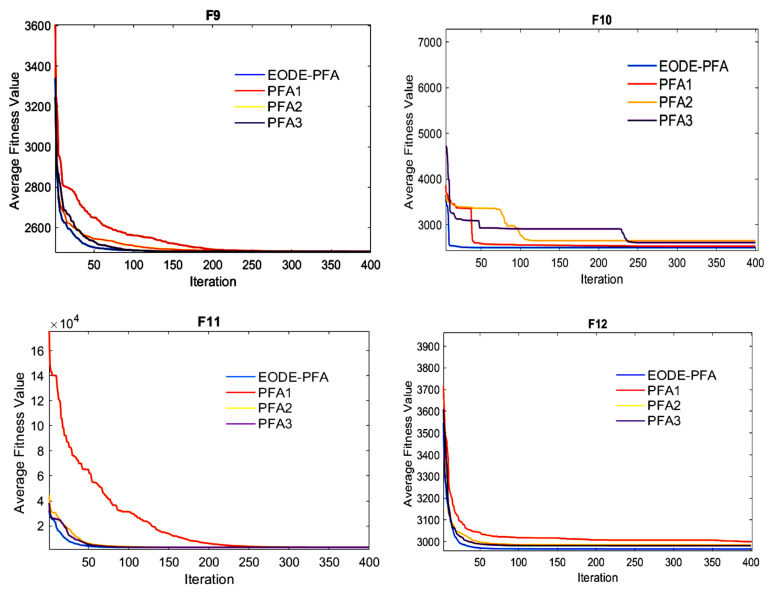
Convergence curves of all algorithms in ablation experiments.

The statistical results of EODE-PFA and the three variants with a single improved strategy removed are shown in Table 4, and the convergence curves are shown in Figure 3. It can be concluded from Table 4 and Figure 3 that the comprehensive optimization performance of EODE-PFA is significantly superior to all variants, fully confirming the necessity, effectiveness, and synergistic advantages of multi-strategy fusion: In all test functions, the mean value of EODE-PFA is better than that of the three variants, demonstrating superior solution accuracy and stability; the convergence curves show that among the 11 test functions, the optimization result of PFA1 with the elite strategy removed is consistently the worst, and the final solution quality is significantly lower than that of other algorithms, which indicates the importance of the elite strategy; PFA2 with the ECOBL mutation operator removed has insufficient local optimization capability, and PFA3 with the differential mutation operator removed has reduced global exploration efficiency; in adition, no single variant can achieve good comprehensive performance. Although the running time of EODE-PFA is generally higher than that of PFA1, it is basically equivalent to that of PFA2 and PFA3, and the magnitude of performance improvement is far greater than the cost of increased running time. Overall, these three types of improved strategies are not simply superimposed but form an efficient synergistic mechanism of “global exploration–local optimization–high-quality solution retention”, which collectively achieves the excellent performance of EODE-PFA, further verifying the effectiveness and necessity of the multi-strategy collaborative design.

### 4.2. Tests on Engineering Optimization Problem

To test the engineering practicality of EODE-PFA, this section applies it to three complex engineering optimization problems with different variable dimensions: three-bar truss design [71], cantilever beam design [72], and welded beam design [73]. Existing relevant studies classify such problems as typical complex optimization problems in the engineering field [1,32,36,39,65,71,74,75], which are classic benchmark problems for verifying the engineering practicality of algorithms. Their complexity lies in the complex optimization characteristics, such as nonlinear constraints, non-convex objectives, and coupling of engineering physical constraints, rather than the number of variables.

Next, we compare EODE-PFA with the eight algorithms mentioned in the previous section. To ensure the reliability of results and fairness of comparison, all algorithms are uniformly set with a population size of 50. The experiments are based on 30 independent runs, with 100 iterations per run. Finally, the mean value of the objective function, the value scheme of each variable, and the average running time of all algorithms are recorded.

#### 4.2.1. Constraint Handling Method

In addition, most engineering optimization problems are subject to constraints. This paper adopts the static penalty function [74] to handle these constraints, with the specific function formula shown in Equation (17). As one of the commonly used constraint handling methods in constrained optimization, the core logic of the static penalty function is to convert the constrained optimization problem into an unconstrained one by imposing penalty terms on solutions that violate the constraints. This form of static penalty function has been widely applied in fields such as structural optimization and engineering parameter optimization [75].

(17) $$\zeta (z)=f(z)\;\pm \;[\sum_{i=1}^{m}l_{i}\;\times \;\max(0,t_{i}(z){)}^{a}+\sum_{j=1}^{m}o_{j}\;\times \;|U_{j}(z)|]$$

where *f*(z) is the original objective function, and ζ(z) is the modified objective function; $l_{i}$ and $o_{j}$ are positive penalty coefficients; ${\max\left(0,t_{i}\left(z\right)\right)}^{\alpha }$ represents the violation degree of inequality constraints, and $\left|U_{j}\left(z\right)\right|$ represents the violation degree of equality constraints. This method assigns a corresponding penalty value to each infeasible solution according to the degree of constraint violation (the more serious the constraint violation, the larger the penalty value), thereby guiding the search individuals to move towards the feasible space.

#### 4.2.2. Three-Bar Truss Design Problem

**Figure 4:**
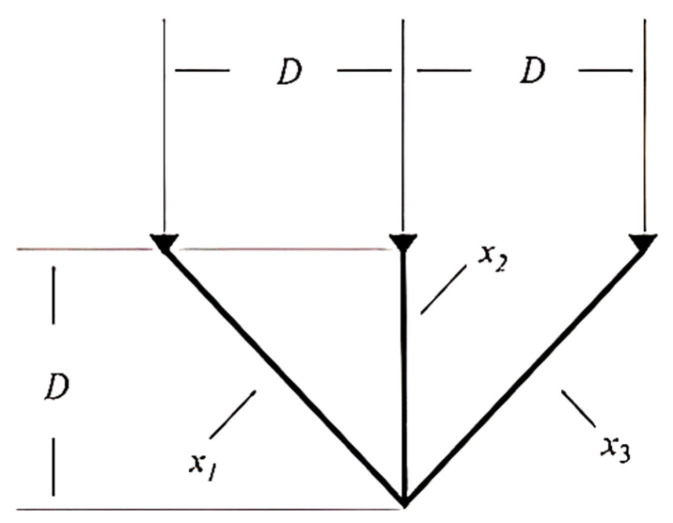
Three-bar truss design.

The first problem is the three-bar truss design problem (TBTD) [71]. Its primary objective is to minimize the total weight of the structure by controlling two parameter variables: $x_{1}$ and $x_{2}=x_{3}$. Here, $x_{1}$ denotes the cross-sectional area of Truss 1 (unit: $m^{2}$), and $x_{2}=x_{3}$ denotes the cross-sectional areas of Truss 2 and Truss 3 (unit: $m^{2}$). The structure and parameters of the three-bar truss are illustrated in Figure 4, and its model is presented as follows:

Minimize

$$f\;\left(x\right)=\left(2\sqrt{2}x_{1}+{\;x}_{2}\right)\;\times \;L,$$

Subject to

$$G_{1}\left(x\right)=\frac{\sqrt{2}x_{1}+x_{2}}{\sqrt{2}{x_{1}}^{2}+2x_{1}x_{2}}\cdot P-\sigma \;\leq 0,$$

$$G_{2}\left(x\right)=\frac{x_{2}}{\sqrt{2}{x_{1}}^{2}+2x_{1}x_{2}}\cdot P-\sigma \;\leq 0,$$

$$G_{3}\left(x\right)=\frac{1}{\sqrt{2}x_{2}+x_{1}}\cdot P-\sigma \;\leq 0,$$

where $0\;\leq \;x_{i}\;\leq \;1,\;i\;=\;1,\;2$, and $x_{1}\;=\;x_{2},$ L = 100 cm, P = 2 kN/$\left(cm^{2}\right)$, σ = 2 kN/$\left(cm^{2}\right)$.

**Figure 5:**
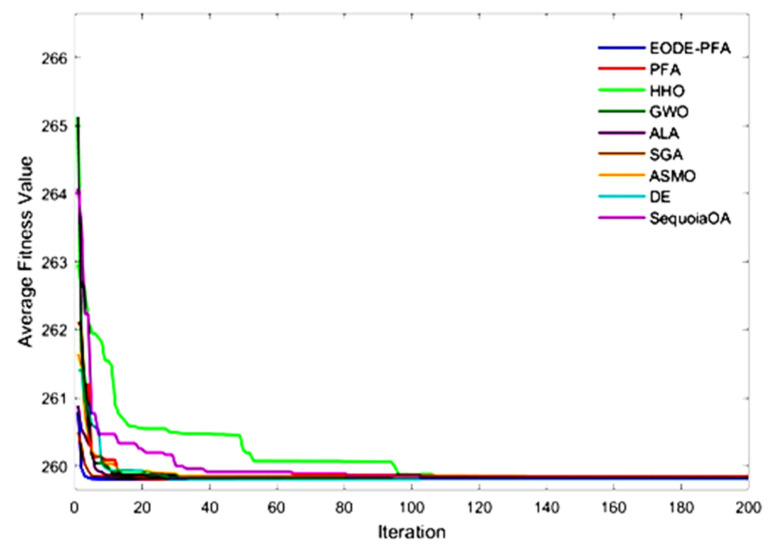
Convergence curves for the three-bar truss design problem optimized by EODE-PFA and other optimizers.

The comparative results between the proposed EODE-PFA and the other eight algorithms are summarized in Table 5, while Figure 5 illustrates the convergence curves of all methods. Evidently, EODE-PFA demonstrates highly satisfactory convergence performance when addressing this problem.

#### 4.2.3. Cantilever Beam Design Problem

The second problem is the cantilever beam design problem (CBD) [72]. Its primary objective is to minimize the weight of the cantilever beam by controlling five variables $x=[x_{1},x_{2},x_{3},x_{4},x_{5}]$, where:

$x_{1}$: Denotes the length of the cantilever beam (m), which determines the overall span of the beam.
$x_{2}$: Denotes the width of the cantilever beam (m), corresponding to the horizontal dimension of the beam’s cross-section.
$x_{3}$: Denotes the height of the cantilever beam (m), referring to the vertical dimension of the beam’s cross-section.
$x_{4}$: Denotes the distance from the acting position of the concentrated load on the beam to the fixed end (m), defining the specific position where the load is applied.
$x_{5}$: Denotes the magnitude of the concentrated load applied on the beam (N), representing the external working load.

**Figure 6:**
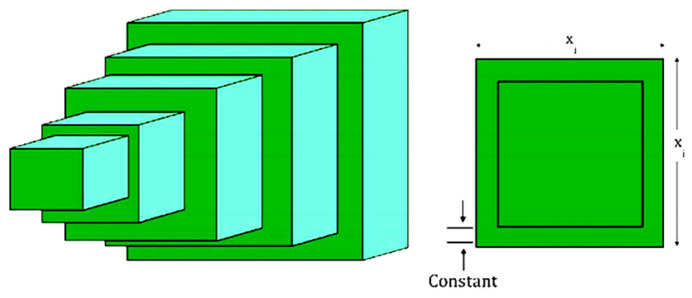
Cantilever beam design.

The structure and parameters of the cantilever beam are illustrated in Figure 6, and its model is presented as follows:

Minimize

$$f(x)\;=\;0.6224\left(x_{1}+\;x_{2}\;+{\;x}_{3}\;+{\;x}_{4\;}+\;x_{5}\right)\;\times L,$$

Subject to

$$G\left(x\right)=\frac{60}{x_{1}^{3}}+\frac{27}{x_{2}^{3}}+\frac{19}{x_{3}^{3}}+\frac{7}{x_{4}^{3}}+\frac{1}{x_{5}^{3}}-1\leq 0,$$

where $0.01\;\leq \;x_{i}\;\leq \;100,\;i\;=\;1,\;2,\;3,\;4,\;5$.

**Figure 7:**
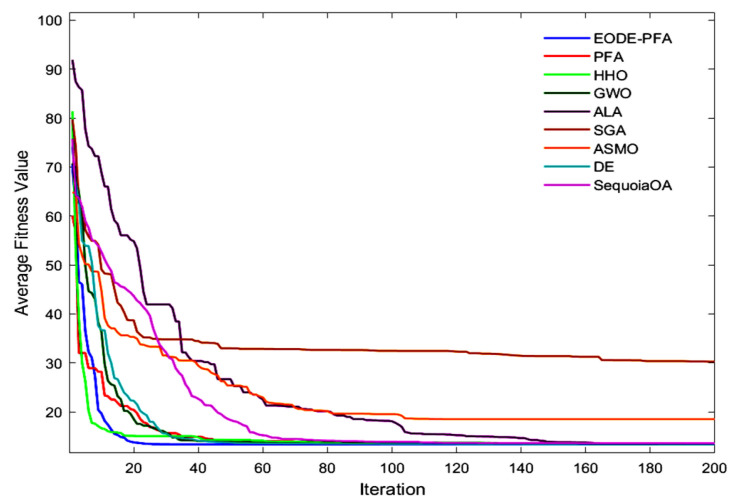
Convergence curves for cantilever beam design problem optimized by EODE-PFA and other optimizers.

The comparative results between the proposed EODE-PFA and the other eight algorithms are summarized in Table 6. Furthermore, Figure 7 reveals that EODE-PFA converges to a favorable solution at the earliest stage.

#### 4.2.4. Welded Beam Design Problem

**Figure 8:**
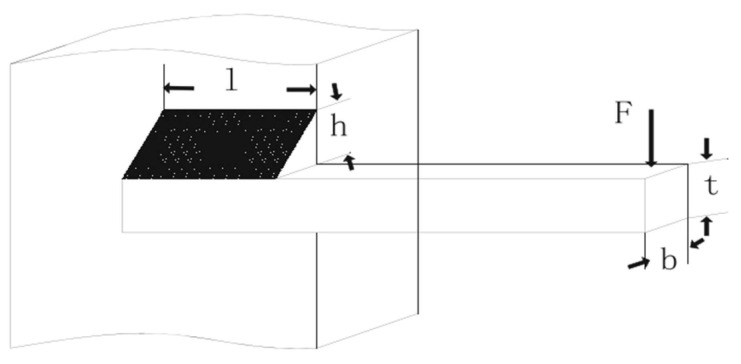
Welded beam design.

The third problem is the welded beam design problem (WBD) [73], which involves four parameter variables: the thickness (t), height (*h*), thickness (*b*), and length (*l*) of the beam rod. The corresponding variable vector is $x=\left(x_{1},\;x_{2},\;x_{3},\;x_{4}\right)=\left(h,\;t,\;b,\;l\right)$. The main objective of this problem is to minimize the total cost of materials, forming, and welding for the cylindrical container. The structure and parameters of the welded beam are illustrated in Figure 8, and its model is presented as follows:

Minimize

$$f(x)=1.10471x_{1}^{2}x_{2}+0.04811x_{3}x_{4}(14.0+x_{2}),$$

Subject to

$$G_{1}(x)=\tau (x)-\tau_{\max}\leq 0,$$

$$G_{2}(x)=\sigma (x)-\sigma_{\max}\leq 0,$$

$$G_{3}(x)=\delta (x)-\delta_{\max}\leq 0,$$

$$G_{4}(x)=x_{1}-x_{4}\leq 0,$$

$$G_{5}(x)=P-P_{c}(x)\leq 0,$$

$$G_{6}(x)=0.125-x_{1}\leq 0,$$

$$G_{7}(x)=1.10471x_{1}^{2}+0.04811x_{3}x_{4}(14.0+x_{2})-5.0\leq 0,$$

where $\tau (x)=\sqrt{(\tau^{\prime }{)}^{2}+2\tau^{\prime }\tau^{\prime \prime }\frac{x_{2}}{2R}+(\tau^{\prime \prime }{)}^{2}}$, $\tau^{\prime }=$ $\frac{P}{\sqrt{2}x_{1}x_{2}}$, $\tau^{\prime \prime }=$ $\frac{MR}{J}$, $M=P\left(L+\frac{x_{2}}{2}\right)$, R= $\sqrt{\frac{x_{2}^{2}}{4}+{\left(\frac{x_{1}+x_{3}}{2}\right)}^{2}}$, $J=2\left(\sqrt{2}x_{1}x_{2}\left(\frac{x_{2}^{2}}{12}+{\left(\frac{x_{1}+x_{3}}{2}\right)}^{2}\right)\right)$, σ(x)= $\frac{6PL}{x_{3}^{2}x_{4}}$, δ(x)= $\frac{4PL^{3}}{Ex_{3}^{3}x_{4}}$, $P_{c}(x)=\frac{4.013E\sqrt{\frac{x_{3}^{2}x_{4}^{6}}{36}}}{L^{2}}\left(1-\frac{x_{3}}{2L}\sqrt{\frac{E}{4G}}\right)$,$P=6000\;\mathrm{lb},\;L=14\;\mathrm{in},\;\delta_{\max}=0.25\;\mathrm{in},\;E=30\times 10^{6}\;\mathrm{psi},\;G=12\times 10^{6}\;\mathrm{psi},\;\tau_{\max}=13,600\;\mathrm{psi},\;\sigma_{\max}=30,000\;\mathrm{psi}$.

**Figure 9:**
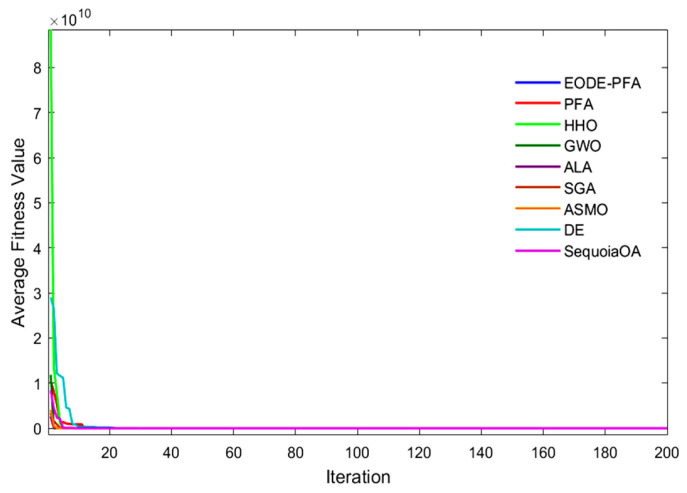
Convergence curves for the welded beam design problem optimized by EODE-PFA and other optimizers.

The comparison results between the proposed EODE-PFA and the other eight algorithms are presented in Table 7. Additionally, Figure 9 indicates that EODE-PFA outperforms others by converging to an optimal solution at the earliest phase.

#### 4.2.5. Performance Evaluation

In the above three complex engineering optimization problems, the performance test results of EODE-PPFA and the remaining eight comparison algorithms are shown in Table 5, Table 6 and Table 7. To intuitively demonstrate the optimization capability of each algorithm, the minimum values of the objective functions are bolded. The comparison diagrams of convergence curves are displayed in Figure 5, Figure 7 and Figure 9.

From the above test results, it can be concluded that EODE-PPFA achieves the optimal objective function values with fast convergence speed in three types of complex engineering optimization problems, namely three-bar truss design, cantilever beam design, and welded beam design. This fully demonstrates the advantages and competitiveness of EODE-PPFA in complex constrained engineering optimization scenarios, indicating its engineering applicability. Meanwhile, the average running time of this algorithm ranks at a medium level among all comparative algorithms, which enables it to effectively balance optimization accuracy and computational efficiency. In addition, the average running time of the proposed algorithm ranks at a moderate level among all comparative algorithms. Although EODE-PFA is slightly slower in terms of running time, the minor time cost it sacrifices is exchanged for optimization accuracy and solution stability that far surpass those of the comparative algorithms. In complex engineering problems, a slight improvement in the optimal objective function value can sometimes bring significant engineering benefits and cost savings, which further confirms the engineering practicality of the proposed algorithm.

## 5. Application of EODE-PFA in Feature Selection

### 5.1. Background

Feature selection is a core preprocessing step for datasets in machine learning. These datasets are derived from real-world scenarios across various fields and typically contain a large number of features, with the feature dimension ranging from thousands to tens of thousands; however, only a subset of these features has a strong correlation with the target [76]. If all features are used in the modeling process, it will not only significantly increase the complexity and training cost of the model but also may lead to model overfitting. Based on this, it is highly necessary to perform feature selection on the dataset prior to modeling. By removing redundant features and noise interference through feature selection, the computational burden of model training can be reduced, and the interpretability and reliability of engineering decision-making models can also be improved [76].

Specifically, the objective of feature selection is to identify and eliminate ineffective features from such real-world high-dimensional datasets and select a subset of impactful features so as to achieve performance comparable to or even superior to that of models trained with the full set of features. Currently, the commonly used feature selection methods in academia are classified into three categories: filter, wrapper, and embedded methods [77], with their specific differences shown in Table 8. The wrapper method is adopted to solve the feature selection problem in the experiments of this section.

### 5.2. Exploitation and Exploration Analysis

As a class of NP-hard problems, feature selection is characterized by both high challenge and high demand, along with high computational cost.

Owing to the advantages of swarm intelligence optimization algorithms, such as flexibility, simplicity, gradient-free property, and decoupling from optimization problems, researchers have begun to combine them with feature selection methods to address the aforementioned issues. For instance, Bing X et al. first applied the multi-objective Particle Swarm Optimization (PSO) algorithm to feature selection tasks in 2012 [77]. The integration of intelligent optimization algorithms with feature selection methods enables the automatic identification of optimal feature subsets, thereby reducing computational complexity and enhancing the interpretability of results. Based on this, this paper applies the proposed EODE-PFA to the feature selection problem.

Feature selection is essentially a multi-objective optimization problem: minimizing the size of the feature subset |S| and maximizing the classifier accuracy Acc(S), where S denotes the optimal feature subset composed of selected features. In this study, we convert it into a single-objective optimization problem by introducing a weight parameter and weighted summation. The objective of this experiment is to reduce the number of features to improve model performance. Consistent with Reference [78], we set ω=0.95. The specific fitness function is given in Equation (18) [78]:

(18) $$F(S)=\omega \cdot \frac{\left|S\right|}{D}+(1-\omega )\times (1-\mathrm{Acc}(S)),\omega =0.95$$

where D is the total number of original features in the dataset; |S| is the number of elements in the feature subset S (i.e., the total number of selected features); Acc(S) represents the classification accuracy of the classifier based on the feature subset S; 1−Acc(S) is the classification error rate based on S; and ω∈[0,1] is a weight coefficient, which is used to adjust the priority between the number of features and the classifier error rate.

EODE-PFA and PFA were originally designed for continuous space optimization, while feature selection is essentially a binary decision problem—specifically, a feature is marked as 1 if selected and 0 if removed. Therefore, before applying EODE-PFA to the feature selection task, it is necessary to convert the continuous search results of the algorithm into binary feature subsets. In the experiments of this section, the threshold truncation mapping method is adopted to perform binary encoding on EODE-PFA. For the continuous position vector component $x_{ij}^{cont}$ of the *i*-th individual generated during the iteration of EODE-PFA, its value is distributed in the range [0,1] after Z-score standardization, and we determine whether a feature is selected by a preset threshold τ. If $x_{ij}^{cont}>\tau$, the feature is selected (encoded as 1); otherwise, it is not selected (encoded as 0). This completes the mapping from continuous search results to binary feature subsets. Reference [79] indicates that setting τ=0.5 can balance the compactness of the feature subset and the classification performance, with the specific conversion formula as follows:

(19) $$x_{ij}^{bin}=\left\{\begin{array}{c} 1,x_{ij}^{cont}>\tau \\ 0,otherwise \end{array}\right.$$

where τ is the preset threshold, representing the criterion for determining whether a feature is selected; $x_{ij}^{cont}$ denotes the *j*-th component of the continuous position vector of the *i*-th individual generated during the iteration of the EODE-PFA algorithm; and $x_{ij}^{bin}$ is the encoded binary component.

To realize the adaptation of EODE-PFA to the feature selection problem, we regard the continuous position vector of each individual in the population as a candidate feature subset, and the continuous position of the leader, after encoding, is the optimal feature subset of the current iteration. The followers, in turn, correspond to the candidate subsets to be optimized. Through an iterative process consisting of leader updating, follower updating, fitness evaluation, and optimal feature subset selection, the size and classification performance of the feature subsets are optimized progressively.

Subsequently, we continued to select the eight algorithms from the previous section and combined them with the wrapper method for comparison. To prevent overfitting, a 5-fold cross-validation strategy was adopted for training and testing in the experiment [80], based on 30 independent samples. The number of iterations per run was set to 60, and the population size *N* was uniformly fixed at 100 (consistent across all algorithms). The experimental setup was kept consistent with that described in the previous section. Finally, the average statistical measurements from 30 independent runs were collected for evaluation.

During this process, the learner was responsible for modeling and evaluating the performance of the dataset after feature selection, so as to compare the effectiveness of feature subsets screened by different algorithms. This study used the K-Nearest Neighbor (KNN) classifier to evaluate the classification accuracy of the fitness function, and K = 3 or K = 5 was selected based on the number of features in the dataset.

In terms of datasets, to verify the performance advantages of EODE-PFA under different feature spaces, this study selected a total of six classic classification datasets, categorized into low-dimensional, medium-dimensional, and high-dimensional types, from the UCI Machine Learning Repository. These datasets exhibit differentiated distributions in terms of sample size, feature dimension, and task scenarios, which can effectively test the ability of each algorithm to screen optimal feature subsets under different data distributions. Detailed information about these datasets is listed in Table 9. Before conducting feature selection on these UCI datasets, we first preprocessed them using Python 3.11 in the Visual Studio Code (VS Code) 1.85 development environment, including basic operations such as identification and handling of missing values, numerical encoding of categorical features, and Z-score standardization of numerical features.

### 5.3. Performance Evaluation and Convergence Curve Analysis

**Figure 10:**
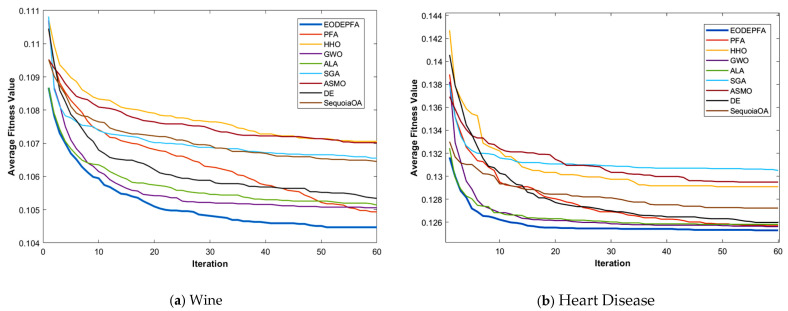

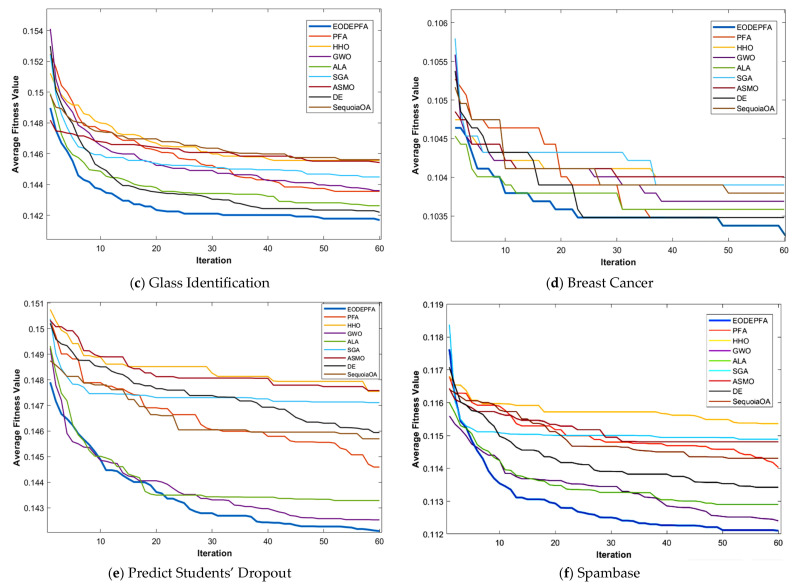
Convergence curves of nine algorithms on six datasets.

The convergence curves of various algorithms on different datasets are presented in Figure 10, and the average performance metrics are listed in Table 10. The results from Figure 10 and Table 10 demonstrate that EODE-PFA outperforms all other algorithms across all test datasets: its average optimal feature number is significantly smaller than that of all comparative algorithms, its average fitness is better, and its average precision is the highest, showing a distinct advantage in balancing feature dimensionality reduction and classification performance.

Meanwhile, compared with PFA, the EODE-PFA, integrated with multi-strategy collaborative improvements achieves an average reduction of 34.2% in the number of optimal selected features, an average optimization of 0.15% in the average fitness, and an average improvement of 5.8% in the average accuracy. This reflects EODE-PFA’s ability to eliminate feature redundancy while retaining key information, providing an efficient and feasible solution for feature selection in large-scale datasets.

## 6. Conclusions

PFA is a bionic intelligent optimization algorithm inspired by animal populations, which has inherent limitations in balancing global exploration and local exploitation capabilities. To address this defect, this paper proposes an enhanced variant, EODE-PFA, which effectively improves the performance of PFA through the collaborative improvement and deep integration of multiple strategies. These improvement strategies are as follows: the elite strategy, the elite opposition-based learning mutation operator centered on elites, the adaptive parameter adjustment mechanism, and the differential mutation operator.

To verify the performance of EODE-PFA, experiments were first conducted on 12 CEC2022 benchmark test functions and three types of complex engineering optimization problems. In this process, to highlight the synergistic effect and overall gain of the multi-strategies proposed in this paper, ablation experiments were carried out on three key strategies. The results show that compared with the original PFA and various intelligent optimization algorithms, EODE-PFA achieves significant improvements in both convergence speed and optimization accuracy, which confirms the rationality and effectiveness of the proposed improvement strategies. Subsequently, based on the wrapper feature selection method, we selected six classification datasets with different dimensions from the UCI Machine Learning Repository and compared EODE-PFA with eight swarm intelligence algorithms in the discrete scenario. Evaluations using the KNN classifier show that EODE-PFA can select feature subsets with optimal sizes while achieving the highest average fitness and classification accuracy. This indicates that EODE-PFA can still effectively balance exploration and exploitation in discrete scenarios and efficiently solve high-dimensional feature selection problems.

In summary, EODE-PFA not only improves the optimization performance of the original algorithm in continuous space but also provides a feasible solution for discrete feature selection, demonstrating excellent engineering applicability and universality. In future research, we plan to apply EODE-PFA to more discrete real-world problems such as engineering scheduling, path planning, and resource allocation.

## Figures and Tables

**Table 1:** The overview of the CEC2022 benchmark test functions [64].

	No.	Functions	$F_{i}^{*}$
Unimodal Function	1	Shifted and full Rotated Zakharov Function	300
Basic Functions	2	Shifted and full Rotated Rosenbrock’s Function	400
	3	Shifted and full Rotated Expanded Schaffer’s F6 Function	600
	4	Shifted and full Rotated Non-Continuous Rastrigin’s Function	800
	5	Shifted and full Rotated Levy Function	900
Hybrid Functions	6	Hybrid Function 1 (N=3)	1800
	7	Hybrid Function 2 (N=6)	2000
	8	Hybrid Function 3 (N=5)	2200
Composition Functions	9	Composition Function 1 (N=5)	2300
	10	Composition Function 2 (N=4)	2400
	11	Composition Function 3 (N=5)	2600
	12	Composition Function 4 (N=6)	2700
Search range: [−100,100]			

**Table 2:** Statistical results of all algorithms in the CEC2022 benchmark set.

*Fun.*	Index	EODE-PFA	PFA	HHO	GWO	ALA	SGA	AS MO	DE	Sequo-iaOA
$F_{1}$	**Best**	**2.23 ×** **10^3^**	6.19 × 10^3^	2.28 × 10^3^	1.73 × 10^4^	6.95 × 10^3^	2.27 × 10^3^	1.92 × 10^4^	2.54 × 10^4^	1.65 × 10^3^
	**Worst**	**9.99 ×** **10^3^**	3.56 × 10^4^	1.23 × 10^4^	4.19 × 10^4^	2.85 × 10^4^	1.23 × 10^4^	5.59 × 10^4^	6.33 × 10^4^	1.80 × 10^4^
	**Mean**	**5.89 ×** **10^3^**	1.65 × 10^4^	6.85 × 10^3^	2.73 × 10^4^	1.55 × 10^4^	6.66 × 10^3^	4.16 × 10^4^	4.74 × 10^4^	8.19 × 10^3^
	**Std**	**1.71 ×** **10^3^**	6.30 × 10^3^	4.90 × 10^3^	5.35 × 10^3^	4.82 × 10^3^	2.77 × 10^3^	8.67 × 10^3^	8.67 × 10^3^	3.06 × 10^3^
	**Time(s)**	0.421	0.174	0.465	0.298	0.361	0.225	0.298	1.139	0.235
$F_{2}$	**Best**	**4.04 ×** **10^2^**	4.45 × 10^2^	4.50 × 10^2^	4.69 × 10^2^	4.08 × 10^2^	4.53 × 10^2^	4.96 × 10^2^	5.03 × 10^2^	4.53 × 10^2^
	**Worst**	**4.75 ×** **10^2^**	5.78 × 10^2^	5.98 × 10^2^	6.57 × 10^2^	5.74 × 10^2^	6.38 × 10^2^	1.00 × 10^3^	5.85 × 10^2^	5.45 × 10^2^
	**Mean**	**4.52 ×** **10^2^**	4.67 × 10^2^	5.12 × 10^2^	5.47 × 10^2^	4.55 × 10^2^	5.07 × 10^2^	6.30 × 10^2^	5.43 × 10^2^	4.69 × 10^2^
	**Std**	**1.53 ×** **10^1^**	3.00 × 10^1^	4.66 × 10^1^	4.84 × 10^1^	3.10 × 10^1^	4.81 × 10^1^	1.15 × 10^2^	2.10 × 10^1^	1.91 × 10^1^
	**Time(s)**	0.439	0.153	0.376	0.305	0.399	0.247	0.351	1.185	0.27
$F_{3}$	**Best**	**2.23 ×** **10^3^**	6.19 × 10^3^	2.28 × 10^3^	1.73 × 10^4^	6.95 × 10^3^	2.27 × 10^3^	1.92 × 10^4^	2.54 × 10^4^	1.65 × 10^3^
	**Worst**	9.99 × 10^3^	3.56 × 10^4^	1.23 × 10^4^	4.19 × 10^4^	2.85 × 10^4^	1.23 × 10^4^	5.59 × 10^4^	6.33 × 10^4^	1.80 × 10^4^
	**Std**	**1.71 ×** **10^3^**	6.30 × 10^3^	4.90 × 10^3^	5.35 × 10^3^	4.82 × 10^3^	2.77 × 10^3^	8.67 × 10^3^	8.67 × 10^3^	3.06 × 10^3^
	**Mean**	5.89 × 10^3^	1.65 × 10^4^	6.85 × 10^3^	2.73 × 10^4^	1.55 × 10^4^	6.66 × 10^3^	4.16 × 10^4^	4.74 × 10^4^	8.19 × 10^3^
	**Time(s)**	0.421	0.174	0.465	0.298	0.361	0.225	0.298	1.139	0.235
$F_{4}$	**Best**	**8.11 ×** **10^2^**	8.26 × 10^2^	8.54 × 10^2^	8.62 × 10^2^	8.28 × 10^2^	8.36 × 10^2^	8.38 × 10^2^	9.12 × 10^2^	8.19 × 10^2^
	**Worst**	**8.52 ×** **10^2^**	8.95 × 10^2^	9.27 × 10^2^	9.72 × 10^2^	9.03 × 10^2^	9.60 × 10^2^	9.55 × 10^2^	9.73 × 10^2^	8.53 × 10^2^
	**Mean**	**8.25 ×** **10^2^**	8.57 × 10^2^	8.84 × 10^2^	9.04 × 10^2^	8.56 × 10^2^	8.88 × 10^2^	9.09 × 10^2^	9.53 × 10^2^	8.37 × 10^2^
	**Std**	9.12 × 10^0^	1.80 × 10^1^	1.60 × 10^1^	2.88 × 10^1^	1.91 × 10^1^	2.52 × 10^1^	3.50 × 10^1^	1.28 × 10^1^	8.63 × 10^0^
	**Time(s)**	0.421	0.174	0.465	0.298	0.361	0.225	0.298	1.139	0.235
$F_{5}$	**Best**	9.01 × 10^2^	9.22 × 10^2^	1.91 × 10^3^	1.40 × 10^3^	9.24 × 10^2^	1.29 × 10^3^	1.04 × 10^3^	9.48 × 10^2^	9.00 × 10^2^
	**Worst**	**9.03 ×** **10^2^**	1.35 × 10^3^	3.14 × 10^3^	3.23 × 10^3^	2.13 × 10^3^	3.78 × 10^3^	1.89 × 10^3^	1.15 × 10^3^	9.05 × 10^2^
	**Mean**	**9.02 ×** **10^2^**	1.03 × 10^3^	2.65 × 10^3^	2.23 × 10^3^	1.27 × 10^3^	2.37 × 10^3^	1.31 × 10^3^	1.02 × 10^3^	9.55 × 10^2^
	**Std**	**4.88 ×** **10^−1^**	1.09 × 10^2^	3.62 × 10^2^	5.11 × 10^2^	2.74 × 10^2^	5.59 × 10^2^	2.60 × 10^2^	5.29 × 10^1^	1.22 × 10^1^
	**Time(s)**	0.533	0.234	0.6	0.371	0.49	0.308	0.337	1.192	0.178
$F_{6}$	**Best**	**1.96 ×** **10^3^**	2.18 × 10^3^	7.52 × 10^3^	3.54 × 10^4^	2.10 × 10^3^	1.97 × 10^3^	2.09 × 10^3^	5.60 × 10^5^	1.38 × 10^4^
	**Worst**	**5.87 ×** **10^3^**	2.02 × 10^4^	1.45 × 10^5^	1.64 × 10^7^	2.53 × 10^4^	2.51 × 10^4^	1.49 × 10^6^	5.77 × 10^6^	6.18 × 10^4^
	**Mean**	**3.84 ×** **10^3^**	8.53 × 10^3^	5.48 × 10^4^	2.16 × 10^6^	1.63 × 10^4^	5.96 × 10^3^	1.09 × 10^5^	2.59 × 10^6^	2.71 × 10^4^
	**Std**	**1.20 ×** **10^3^**	7.34 × 10^3^	3.33 × 10^4^	3.86 × 10^6^	8.45 × 10^3^	6.00 × 10^3^	3.76 × 10^5^	9.92 × 10^5^	9.39 × 10^3^
	**Time(s)**	1.227	0.921	2.534	1.462	2.002	0.678	0.767	2.941	0.683
$F_{7}$	**Best**	2.04 × 10^3^	2.05 × 10^3^	2.12 × 10^3^	2.09 × 10^3^	2.03 × 10^3^	2.09 × 10^3^	2.05 × 10^3^	2.08 × 10^3^	2.05 × 10^3^
	**Worst**	**2.08 ×** **10^3^**	2.23 × 10^3^	2.26 × 10^3^	2.12 × 10^3^	2.17 × 10^3^	2.28 × 10^3^	2.20 × 10^3^	2.10 × 10^3^	2.10 × 10^3^
	**Mean**	**2.06 ×** **10^3^**	2.14 × 10^3^	2.17 × 10^3^	2.11 × 10^3^	2.08 × 10^3^	2.17 × 10^3^	2.11 × 10^3^	2.09 × 10^3^	2.08 × 10^3^
	**Std**	1.53 × 10^1^	6.68 × 10^1^	6.14 × 10^1^	1.41 × 10^1^	5.55 × 10^1^	7.26 × 10^1^	6.92 × 10^1^	1.03 × 10^1^	2.56 × 10^1^
	**Time(s)**	1.237	0.911	1.48	1.201	1.254	0.705	0.935	2.226	0.384
$F_{8}$	**Best**	**2.22 ×** **10^3^**	2.23 × 10^3^	2.23 × 10^3^	2.23 × 10^3^	2.23 × 10^3^	2.23 × 10^3^	2.22 × 10^3^	2.24 × 10^3^	2.23 × 10^3^
	**Worst**	**2.24 ×** **10^3^**	2.47 × 10^3^	2.45 × 10^3^	2.39 × 10^3^	2.25 × 10^3^	2.48 × 10^3^	2.37 × 10^3^	2.25 × 10^3^	2.24 × 10^3^
	**Mean**	**2.23 ×** **10^3^**	2.28 × 10^3^	2.27 × 10^3^	2.30 × 10^3^	2.23 × 10^3^	2.33 × 10^3^	2.26 × 10^3^	2.25 × 10^3^	2.23 × 10^3^
	**Std**	4.58 × 10^0^	6.77 × 10^1^	6.13 × 10^1^	6.39 × 10^1^	4.64 × 10^0^	6.56 × 10^1^	4.94 × 10^1^	4.09 × 10^0^	1.38 × 10^0^
	**Time(s)**	1.086	0.465	1.278	0.672	1.002	0.701	0.97	1.995	0.456
$F_{9}$	**Best**	**2.48 ×** **10^3^**	2.48 × 10^3^	2.48 × 10^3^	2.50 × 10^3^	2.48 × 10^3^	2.48 × 10^3^	2.48 × 10^3^	2.48 × 10^3^	2.48 × 10^3^
	**Worst**	**2.48 ×** **10^3^**	2.49 × 10^3^	2.57 × 10^3^	2.54 × 10^3^	2.48 × 10^3^	2.67 × 10^3^	2.49 × 10^3^	2.49 × 10^3^	2.49 × 10^3^
	**Mean**	**2.48 ×** **10^3^**	2.48 × 10^3^	2.50 × 10^3^	2.51 × 10^3^	2.48 × 10^3^	2.53 × 10^3^	2.48 × 10^3^	2.49 × 10^3^	2.49 × 10^3^
	**Std**	**2.74 ×** **10^−2^**	1.73 × 10^0^	1.95 × 10^1^	1.41 × 10^1^	6.18 × 10^−2^	5.28 × 10^1^	2.82 × 10^0^	1.55 × 10^0^	2.49 × 10^0^
	**Time(s)**	1.882	0.749	2.606	1.39	1.708	1.246	1.592	4.79	0.799
$F_{10}$	**Best**	**2.50 ×** **10^3^**	2.50 × 10^3^	2.50 × 10^3^	2.50 × 10^3^	2.50 × 10^3^	2.50 × 10^3^	2.50 × 10^3^	2.50 × 10^3^	2.50 × 10^3^
	**Worst**	**2.50 ×** **10^3^**	5.44 × 10^3^	4.46 × 10^3^	6.73 × 10^3^	4.26 × 10^3^	5.59 × 10^3^	4.96 × 10^3^	2.54 × 10^3^	4.75 × 10^3^
	**Mean**	**2.50 ×** **10^3^**	2.85 × 10^3^	3.61 × 10^3^	4.59 × 10^3^	3.06 × 10^3^	3.52 × 10^3^	3.49 × 10^3^	2.51 × 10^3^	2.73 × 10^3^
	**Std**	**1.24 ×** **1** **0^−^** **^1^**	9.13 × 10^2^	8.04 × 10^2^	1.83 × 10^3^	7.47 × 10^2^	1.25 × 10^3^	9.75 × 10^2^	1.28 × 10^1^	7.11 × 10^2^
	**Time(s)**	1.227	0.921	2.534	1.462	2.002	0.678	0.767	2.941	0.683
${F\;}_{11}$	**Best**	**2.90 ×** **10^3^**	2.75 × 10^3^	2.95 × 10^3^	3.98 × 10^3^	2.92 × 10^3^	3.04 × 10^3^	3.01 × 10^3^	2.90 × 10^3^	3.05 × 10^3^
	**Worst**	**2.90 ×** **10^3^**	3.00 × 10^3^	3.60 × 10^3^	5.37 × 10^3^	2.98 × 10^3^	3.70 × 10^3^	5.16 × 10^3^	2.90 × 10^3^	3.22 × 10^3^
	**Mean**	**2.90 ×** **10^3^**	2.99 × 10^3^	3.22 × 10^3^	5.24 × 10^3^	3.00 × 10^3^	3.64 × 10^3^	3.81 × 10^3^	2.90 × 10^3^	3.20 × 10^3^
	**Std**	**7.73 ×** **10^−6^**	6.46 × 10^1^	5.66 × 10^2^	5.95 × 10^2^	6.80 × 10^1^	3.51 × 10^2^	1.82 × 10^3^	2.48 × 10^−2^	7.91 × 10^1^
	**Time(s)**	3.568	1.279	3.02	1.637	1.976	1.449	1.581	2.701	0.36
$F_{12}$	**Best**	**2.93 ×** **10^3^**	2.94 × 10^3^	2.98 × 10^3^	2.98 × 10^3^	2.94 × 10^3^	2.97 × 10^3^	2.94 × 10^3^	2.94 × 10^3^	2.95 × 10^3^
	**Worst**	2.97 × 10^3^	3.10 × 10^3^	3.38 × 10^3^	3.02 × 10^3^	2.95 × 10^3^	3.21 × 10^3^	2.99 × 10^3^	2.96 × 10^3^	3.00 × 10^3^
	**Mean**	**2.95 ×** **10^3^**	2.99 × 10^3^	3.12 × 10^3^	3.00 × 10^3^	2.95 × 10^3^	3.04 × 10^3^	2.96 × 10^3^	2.95 × 10^3^	2.97 × 10^3^
	**Std**	9.43 × 10^0^	3.80 × 10^1^	1.36 × 10^2^	1.21 × 10^1^	4.39 × 10^0^	7.70 × 10^1^	1.65 × 10^1^	4.81 × 10^0^	1.39 × 10^1^
	**Time(s)**	1.424	0.888	1.732	0.797	0.91	0.706	1.19	2.153	0.865

**Table 3:** Wilcoxon signed-rank test results for CEC2022 functions.

*Fun.*	Index	EODE-PFA vs. PFA	EODE-PFA vs. HHO	EODE-PFA vs. GWO	EODE-PFA vs. ALA	EODE-PFA vs. SGA	EODE-PFA vs. ASMO	EODE-PFA vs. DE	EODE-PFA vs. SequoiaOA
$F_{1}$	***p-value***	0.0001	0.0001	0.0001	0.0001	0.0001	0.0001	0.0001	0.4330
	R+	412	390	412	382	322	412	422	45
	R−	53	53	53	53	53	53	53	320
	**sign**	+	+	+	+	+	+	+	−
$F_{2}$	***p-value***	0.0036	0.0007	0.0001	0.0605	0.0003	0.0304	0.0089	0.1259
	R+	380	400	412	20	405	370	370	220
	R−	85	65	53	275	60	195	95	195
	**sign**	+	+	+	−	+	+	+	=
$F_{3}$	***p-value***	0.0001	0.0001	0.0001	0.0279	0.0001	0.0017	0.0003	0.1259
	R+	412	412	412	340	412	390	405	0
	R−	53	53	53	125	53	75	60	195
	**sign**	+	+	+	+	+	+	+	−
$F_{4}$	***p-value***	0.0001	0.0001	0.0001	0.0001	0.0001	0.0001	0.0001	0.0813
	R+	405	395	325	325	412	412	412	35
	R−	50	70	3	300	53	53	53	370
	**sign**	+	+	+	=	+	+	+	−
$F_{5}$	***p-value***	0.0001	0.0001	0.0001	0.0001	0.0001	0.0001	0.0001	0.4330
	R+	412	325	412	325	412	312	412	45
	R−	53	3	53	3	53	20	53	220
	**sign**	+	+	+	+	+	+	+	−
$F_{6}$	***p-value***	0.0033	0.0001	0.0001	0.0002	0.0579	0.0004	0.0001	0.0001
	R+	385	412	412	405	340	400	412	412
	R−	80	53	53	60	325	65	53	53
	**sign**	+	+	+	+	=	+	+	+
$F_{7}$	***p-value***	0.0025	0.0001	0.0001	0.5503	0.0001	0.7369	0.0001	0.0040
	R+	390	412	412	50	412	225	412	375
	R−	75	53	53	235	53	240	53	90
	**sign**	+	+	+	−	+	=	+	+
$F_{8}$	***p-value***	0.0012	0.0001	0.0001	0.0093	0.0001	0.0001	0.0011	0.0090
	R+	395	412	412	365	412	412	395	365
	R−	70	53	53	100	53	53	70	100
	**sign**	+	+	+	+	+	+	+	+
$F_{9}$	***p-value***	0.0039	0.0020	0.0020	0.0020	0.0020	0.0039	0.0020	0.9219
	R+	380	350	390	390	390	380	350	0
	R−	85	125	85	75	75	85	125	233
	**sign**	+	+	+	+	+	+	+	−
$F_{10}$	R+	375	400	412	380	375	335	355	50
	R−	90	65	53	85	90	70	310	215
	sign	+	+	+	+	+	+	+	−
	R+	400	412	412	382	412	412	412	412
$F_{11}$	***p-value***	0.0010	0.0001	0.0001	0.0001	0.0001	0.0001	0.0001	0.0001
	R+	400	412	412	382	412	412	412	412
	R−	385	53	53	53	53	53	53	53
	**sign**	+	+	+	+	+	+	+	+
$F_{12}$	***p-value***	0.0017	0.0020	0.0059	0.0059	0.0137	0.3223	0.0137	0.0919
	R+	390	390	370	370	355	0	55	280
	R−	75	75	95	95	110	210	310	285
	**sign**	+	+	+	+	+	−	+	=

**Table 4:** Statistical results of all algorithms in ablation experiments.

Fun.	Index	EODE- PFA	PFA1	PFA2	PFA3
$F_{1}$	Best	**3.00 × 10^2^**	2.55 × 10^3^	3.00 × 10^2^	4.18 × 10^3^
	Worst	**3.02 × 10^2^**	5.64 × 10^3^	3.12 × 10^2^	7.45 × 10^3^
	Mean	**3.01 × 10^2^**	3.92 × 10^3^	3.04 × 10^2^	6.16 × 10^3^
	Std	**6.80 × 10^−1^**	1.11 × 10^3^	4.78 × 10^0^	1.49 × 10^3^
	time(s)	0.214	0.096	0.209	0.216
$F_{2}$	Best	**4.45 × 10^2^**	4.50 × 10^2^	4.49 × 10^2^	4.49 × 10^2^
	Worst	4.71 × 10^2^	4.78 × 10^2^	4.71 × 10^2^	**4.71 × 10^2^**
	Mean	**4.53 × 10^2^**	4.60 × 10^2^	4.54 × 10^2^	4.58 × 10^2^
	Std	1.06 × 10^1^	1.30 × 10^1^	**9.78 × 10** ^0^	1.14 × 10^1^
	time(s)	0.201	0.095	0.206	0.201
$F_{3}$	Best	6.01 × 10^2^	6.08 × 10^2^	6.01 × 10^2^	**6.00 × 10^2^**
	Worst	**6.04 × 10^2^**	6.19 × 10^2^	6.15 × 10^2^	6.20 × 10^2^
	Mean	**6.02 × 10^2^**	6.13 × 10^2^	6.08 × 10^2^	6.04 × 10^2^
	Std	**1.33 × 10^0^**	4.12 × 10^0^	6.41 × 10^0^	9.05 × 10^0^
	time(s)	0.311	0.159	0.388	0.391
$F_{4}$	Best	**8.12 × 10^2^**	8.40 × 10^2^	8.12 × 10^2^	8.13 × 10^2^
	Worst	**8.23 × 10^2^**	9.42 × 10^2^	8.32 × 10^2^	8.31 × 10^2^
	Mean	**8.19 × 10^2^**	8.81 × 10^2^	8.19 × 10^2^	8.21 × 10^2^
	Std	**3.53 × 10^0^**	3.76 × 10^1^	5.73 × 10^0^	6.21 × 10^0^
	time(s)	0.743	0.327	0.74	0.765
$F_{5}$	Best	**9.01 × 10^2^**	9.07 × 10^2^	9.02 × 10^2^	**9.01 × 10^2^**
	Worst	1.64 × 10^3^	2.45 × 10^3^	1.24 × 10^3^	**1.06 × 10^3^**
	Mean	**9.36 × 10^2^**	1.16 × 10^3^	1.06 × 10^3^	9.97 × 10^2^
	Std	2.28 × 10^2^	4.68 × 10^2^	1.22 × 10^2^	**5.39 × 10^1^**
	time(s)	0.733	0.35	0.774	0.78
$F_{6}$	Best	2.01 × 10^3^	1.21 × 10^5^	**1.95 × 10^3^**	1.95 × 10^3^
	Worst	**5.64 × 10^3^**	6.45 × 10^5^	6.73 × 10^3^	8.50 × 10^3^
	Mean	**3.34 × 10^3^**	4.12 × 10^5^	3.86 × 10^3^	4.66 × 10^3^
	Std	**1.46 × 10^3^**	1.96 × 10^5^	2.16 × 10^3^	2.59 × 10^3^
	time(s)	0.325	0.154	0.323	0.339
$F_{7}$	Best	**2.02 × 10^3^**	2.05 × 10^3^	2.05 × 10^3^	2.04 × 10^3^
	Worst	2.11 × 10^3^	2.21 × 10^3^	**2.08 × 10^3^**	2.08 × 10^3^
	Mean	**2.06 × 10^3^**	2.10 × 10^3^	2.06 × 10^3^	2.07 × 10^3^
	Std	3.40 × 10^1^	6.60 × 10^1^	**1.31 × 10^1^**	1.55 × 10^1^
	time(s)	0.724	0.295	0.760	0.759
$F_{8}$	Best	**2.22 × 10^3^**	2.25 × 10^3^	2.23 × 10^3^	2.23 × 10^3^
	Worst	**2.23 × 10^3^**	2.39 × 10^3^	2.23 × 10^3^	2.23 × 10^3^
	Mean	**2.23 × 10^3^**	2.29 × 10^3^	2.23 × 10^3^	2.23 × 10^3^
	Std	1.57 × 10^0^	5.73 × 10^1^	**9.86 × 10^−1^**	2.09 × 10^0^
	time(s)	0.872	0.341	0.865	0.894
$F_{9}$	Best	**2.48 × 10^3^**	2.48 × 10^3^	2.48 × 10^3^	2.48 × 10^3^
	Worst	**2.48 × 10^3^**	2.48 × 10^3^	2.48 × 10^3^	2.48 × 10^3^
	Mean	**2.48 × 10^3^**	2.48 × 10^3^	2.48 × 10^3^	2.48 × 10^3^
	Std	**2.56 × 10^−2^**	5.89 × 10^−1^	1.32 × 10^−1^	5.13 × 10^−2^
	time(s)	0.745	0.293	0.764	0.748
$F_{10}$	Best	**2.50 × 10^3^**	2.50 × 10^3^	2.50 × 10^3^	2.50 × 10^3^
	Worst	**2.50 × 10^3^**	2.65 × 10^3^	3.10 × 10^3^	2.91 × 10^3^
	Mean	**2.50 × 10^3^**	2.53 × 10^3^	2.65 × 10^3^	2.61 × 10^3^
	Std	**6.49 × 10^−2^**	6.62 × 10^1^	2.61 × 10^2^	1.76 × 10^2^
	time(s)	1.507	0.608	1.479	1.501
$F_{11}$	Best	**2.90 × 10^3^**	2.95 × 10^3^	2.90 × 10^3^	2.90 × 10^3^
	Worst	**2.90 × 10^3^**	2.97 × 10^3^	2.90 × 10^3^	2.90 × 10^3^
	Mean	**2.90 × 10^3^**	2.96 × 10^3^	2.90 × 10^3^	2.90 × 10^3^
	Std	**4.48 × 10^−11^**	6.99 × 10^0^	**3.00 × 10^−6^**	**2.26 × 10^−8^**
	time(s)	0.894	0.348	0.923	0.9
$F_{12}$	Best	2.95 × 10^3^	2.96 × 10^3^	**2.94 × 10^3^**	2.94 × 10^3^
	Worst	3.02 × 10^3^	3.02 × 10^3^	**2.99 × 10^3^**	3.00 × 10^3^
	Mean	**2.96 × 10^3^**	3.00 × 10^3^	2.97 × 10^3^	2.98 × 10^3^
	Std	2.23 × 10^1^	2.36 × 10^1^	**1.87 × 10^1^**	2.64 × 10^1^
	time(s)	0.602	0.231	0.598	0.607

**Table 5:** The comparison results of the three-bar truss design problem.

Algorithm	$x_{1}$	$x_{2}$	Objective Function	AVG. Time(s)
EODEPFA	0.76494	0.39596	**259.80505**	0.1074
PFA	0.76494	0.39596	**259.80505**	0.0559
HHO	0.76440	0.39749	259.80527	0.1406
GWO	0.76482	0.39613	259.80507	0.0611
ALA	0.76494	0.39596	**259.80505**	0.0801
SGA	0.76600	0.39340	259.80582	0.0998
ASMO	0.76494	0.39596	**259.80505**	0.0847
DE	0.76491	0.39605	**259.80505**	0.2687
SequoiaOA	0.76103	0.40410	259.81822	0.0506

**Table 6:** The comparison results of the cantilever beam design problem.

Algorithm	$x_{1}$	$x_{2}$	$x_{3}$	$x_{4}$	$x_{5}$	Objective Function	AVG. Time(s)
EODEPFA	6.01157	5.30525	4.49101	3.49889	2.15107	**13.36026**	0.0781
PFA	6.01247	5.31012	4.48674	3.49934	2.14889	13.36007	0.0457
HHO	5.98145	5.31529	4.42932	3.65457	2.09506	13.37147	0.1059
GWO	5.97370	5.32650	4.49907	3.50086	2.15845	13.36071	0.0485
ALA	6.11907	5.19096	4.45497	3.57338	2.13223	13.36824	0.0733
SGA	6.66611	21.77947	3.55157	5.03495	2.55486	24.63893	0.0824
ASMO	6.68119	5.62442	3.89169	3.30374	2.37999	13.62868	0.0707
DE	5.97347	5.29736	4.56176	3.52794	2.10933	13.62868	0.2601
SequoiaOA	6.01157	5.30525	4.49101	3.49889	2.15107	13.36026	0.0781

**Table 7:** The comparison results of the welded beam design problem.

Algorithm	$x_{1}$	$x_{2}$	$x_{3}$	$x_{4}$	Objective Function	AVG. Time(s)
EODEPFA	0.16871	3.82299	10.00000	0.16800	**1.56166**	0.3583
PFA	0.17098	3.77458	9.98812	0.16858	1.56752	0.1918
HHO	0.15659	4.20597	9.99284	0.16824	1.58648	0.4972
GWO	0.16884	3.83015	10.00000	0.16801	1.56249	0.2117
ALA	0.16877	3.82671	10.00000	0.16800	1.56184	0.2727
SGA	0.14165	4.77481	9.89370	0.17202	1.64311	0.3069
ASMO	0.16876	3.82701	10.00000	0.16800	1.56184	0.2776
DE	0.17670	3.77539	9.74464	0.17711	1.56184	0.8802
SequoiaOA	0.16871	3.82299	10.00000	0.16800	1.56166	0.3583

**Table 8:** Three common categories of feature selection methods.

Feature Selection Method	Selection Approach
Filter Method	Select features by evaluating the statistical correlation between features and the target variable.
Filter Method	Select features by constructing and evaluating multiple models.
Filter Method	Automatically screen features during training through regularization or built-in model mechanisms.

**Table 9:** Six datasets used in simulation experiments.

Dataset	Samples	Original Features	Classes	Task
**Wine**	178	12	3	Wine Origin Classification
**Heart Disease**	303	13	2	Heart Disease Prediction
**Glass Identification**	214	9	6	Glass Type Classification
**Breast Cancer**	569	30	2	Breast Cancer Diagnosis
**Predict Students’ Dropout**	4424	36	3	Student Dropout Risk Prediction (Dropout, Enrolled, Graduated)
**Spambase**	4601	57	2	Spam Email Detection

**Table 10:** Feature selection comparison results of all algorithms for test datasets.

Algorithm	Avg. Feature No.	Avg. Fitness	Avg. Precision
EODE-PFA	5.20	0.1044	0.9858
PFA	6.58	0.1050	0.9543
HHO	7.30	0.1071	0.7897
GWO	6.22	0.1050	0.9554
ALA	7.40	0.1051	0.9555
SGA	7.28	0.1066	0.9452
ASMO	7.62	0.1070	0.9470
DE	8.14	0.1053	0.9508
SequoiaOA	6.90	0.1065	0.9470
(**a**) Wine			
**Algorithm**	**Avg. Feature No.**	**Avg. Fitness**	**Avg. Precision**
EODE-PFA	4.23	0.1256	0.9589
PFA	5.36	0.1259	0.9260
HHO	7.53	0.1291	0.6410
GWO	5.57	0.1258	0.8655
ALA	6.21	0.1258	0.8136
SGA	5.53	0.1305	0.8521
ASMO	6.57	0.1295	0.7987
DE	5.87	0.1260	0.8312
SequoiaOA	5.42	0.1272	0.9066
(**b**) Heart Disease			
**Algorithm**	**Avg. Feature No.**	**Avg. Fitness**	**Avg. Precision**
EODE-PFA	3.82	0.1417	0.9605
PFA	5.20	0.1436	0.8727
HHO	5.78	0.1456	08584
GWO	7.36	0.1436	0.6513
ALA	4.43	0.1426	0.8954
SGA	4.06	0.1445	0.9334
ASMO	7.02	0.1454	0.7387
DE	7.19	0.1422	0.7324
SequoiaOA	6.59	0.1456	0.7232
(**c**) Glass Identification			
**Algorithm**	**Avg. Feature No.**	**Avg. Fitness**	**Avg. Precision**
EODE-PFA	12.67	0.1033	0.9849
PFA	14.33	0.1035	0.9409
HHO	14.60	0.1040	0.9464
GWO	15.73	0.1037	0.9279
ALA	18.33	0.1036	0.8653
SGA	16.33	0.1039	0.8970
ASMO	17.33	0.1040	0.8561
DE	18.67	0.1035	0.8634
SequoiaOA	12.93	0.1038	0.9696
(**d**) Breast Cancer			
**Algorithm**	**Avg. Feature No.**	**Avg. Fitness**	**Avg. Precision**
EODE-PFA	7.00	0.1421	0.9684
PFA	14.00	0.1446	0.9121
HHO	22.20	0.1475	0.8654
GWO	9.40	0.1425	0.9543
ALA	13.40	0.1433	0.9077
SGA	13.80	0.1471	0.9220
ASMO	18.80	0.1476	0.8780
DE	21.40	0.1459	0.8814
SequoiaOA	15.60	0.1457	0.9092
(**e**) Predict Students’ Dropout			
**Algorithm**	**Avg. Feature No.**	**Avg. Fitness**	**Avg. Precision**
EODE-PFA	18.60	0.1121	0.9793
PFA	30.20	0.1140	0.8700
HHO	32.20	0.1154	0.8394
GWO	27.80	0.1124	0.9050
ALA	32.00	0.1129	0.8956
SGA	48.40	0.1149	0.8897
ASMO	33.60	0.1148	0.9073
DE	40.80	0.1134	0.9180
SequoiaOA	30.80	0.1143	0.8831
(**f**) Spambase			

## Data Availability

All data generated or analyzed during this study are included in this published article. The datasets analyzed in this study are available in the UCI Machine Learning Repository, with the specific links provided as follows: • Glass Identification: https://archive.ics.uci.edu/dataset/42/glass+identification, accessed on 1 December 2025. • Spambase: https://archive.ics.uci.edu/ml/datasets/Spambase, accessed on 1 December 2025. • Heart Disease: https://archive.ics.uci.edu/dataset/45/heart+disease, accessed on 1 December 2025. • Wine: https://archive.ics.uci.edu/dataset/109/wine, accessed on 1 December 2025. • Breast Cancer: https://archive.ics.uci.edu/dataset/17/breast+cancer+wisconsin+diagnostic, accessed on 1 December 2025. • Predict Students’ Dropout: https://archive.ics.uci.edu/dataset/697/predict+students+dropout+and+academic+success, accessed on 1 December 2025.
